# Supplementary material for: Suppression of Vps13 adaptor protein mutants reveals a central role for PI4P in regulating prospore membrane extension
Source: PLoS Genet. 2021 Aug 18;17(8):e1009727. doi: 10.1371/journal.pgen.1009727 (PMC8372973; doi:10.1371/journal.pgen.1009727)
Supplement: S1 Fig — (A) Assessment of Stt4 activity. stt4-4 (AAY102) was transformed indicated plasmid, and grown on SD plates for 2 days at permissive (30°C) or nonpermissive (37°C) temperature. (B) Assessment of sporulation in spo73Δ (TC545) overexpressing wild-type STT4 (WT) or STT4-KD. More than 200 cells were observed in three independent colonies of each strain harboring indicated plasmids (for a total of > 600 cells). The bar graph shows the mean of the percentage of asci (N = 3). (C and D) Localization of GFPEnvy-Ypp1 (C) and Sfk1-GFP (D) in wild-type (AN120) or spo73Δ (TC545) cells during PSM formation. mK, mKate2. mKate2-Spo2051–91, a PSM marker. Scale bar, 5 μm. (PDF) [file pgen.1009727.s001.pdf]

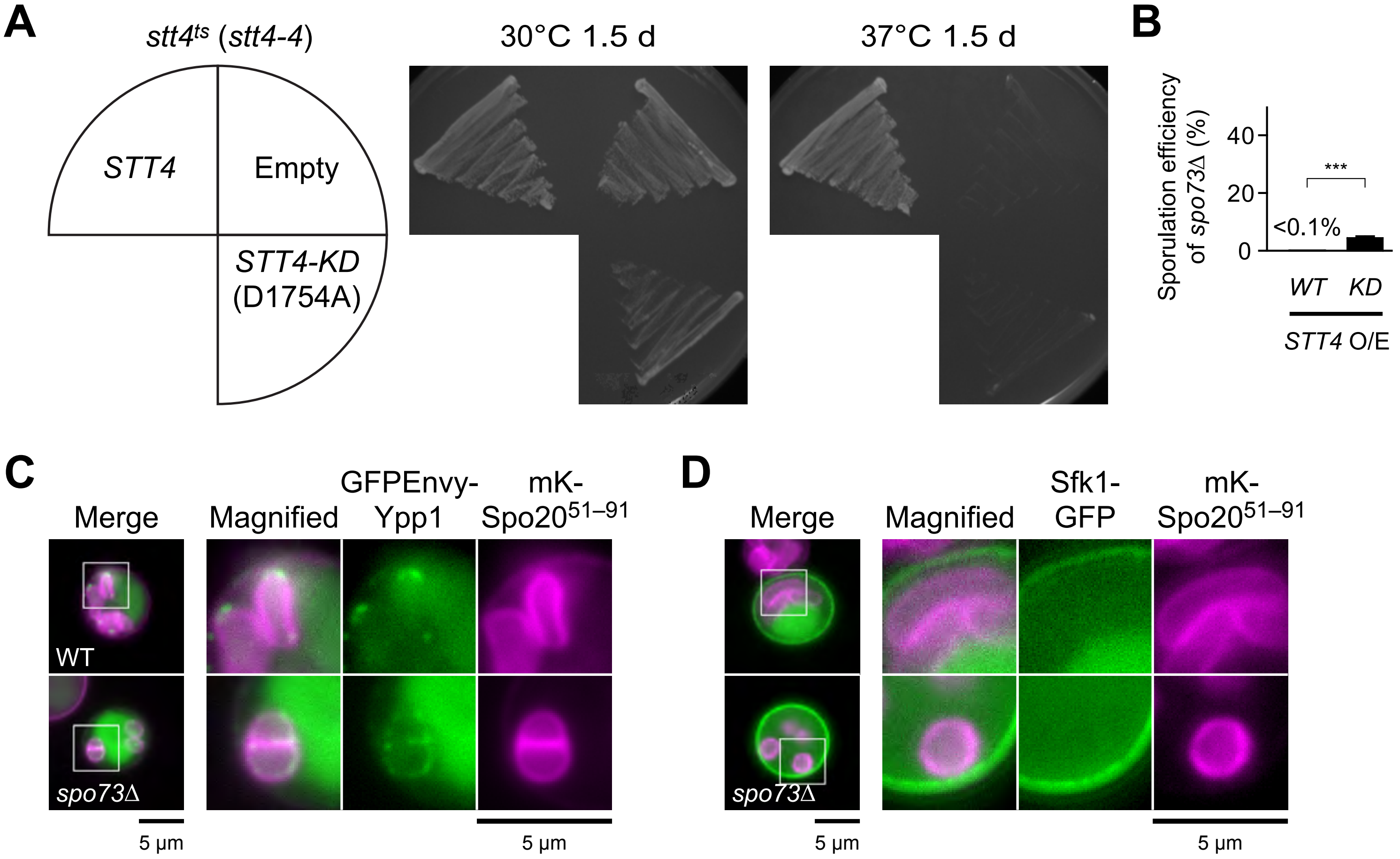
A
stt4ts (stt4-4)
STT4
Empty
STT4-KD
(D1754A)
30°C 1.5 d
37°C 1.5 d
B
Sporulation efficiency of spo73Δ (%)
40
20
0
***
<0.1%
WT
KD
STT4 O/E
C
Merge
Magnified
GFPEnvy-Ypp1
mK-Spo20 51–91
WT
spo73Δ
5 μm
5 μm
D
Merge
Magnified
Sfk1-GFP
mK-Spo20 51–91
spo73Δ
5 μm
5 μm

S1 Fig.

S1 Fig. The PI4K complex localizes on the PSM, and suppression occurs through a dominant-negative effect.

(A) Assessment of Stt4 activity. *stt4-4* (AAY102) was transformed indicated plasmid, and grown on SD plates for 2 days at permissive (30°C) or nonpermissive (37°C) temperature. (B) Assessment of sporulation in *spo73*Δ (TC545) overexpressing wild-type *STT4* (WT) or *STT4-KD*. More than 200 cells were observed in three independent colonies of each strain harboring indicated plasmids (for a total of > 600 cells). The bar graph shows the mean of the percentage of asci (N = 3). (C and D) Localization of GFPEnvy-Ypp1 (C) and Sfk1-GFP (D) in wild-type (AN120) or *spo73*Δ (TC545) cells during PSM formation. mK, mKate2. mKate2-Spo20$^{51–91}$, a PSM marker. Scale bar, 5 µm.
