# Supplementary material for: Suppression of Vps13 adaptor protein mutants reveals a central role for PI4P in regulating prospore membrane extension
Source: PLoS Genet. 2021 Aug 18;17(8):e1009727. doi: 10.1371/journal.pgen.1009727 (PMC8372973; doi:10.1371/journal.pgen.1009727)
Supplement: S2 Fig — Localization of Sac12–517 chimera proteins in spo73Δ (TC545) cells during PSM formation. mR, mRFP. PD, phosphatase-dead. Scale bar, 5 μm. (PDF) [file pgen.1009727.s002.pdf]

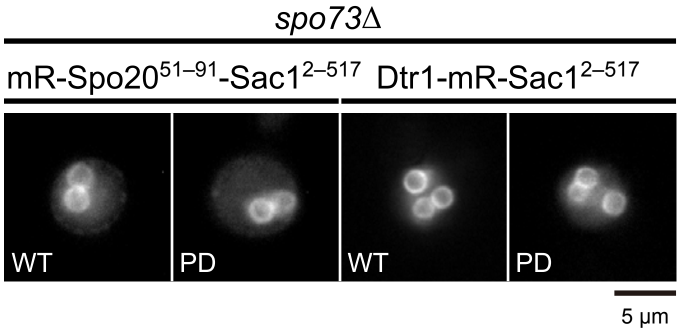
spo73Δ
mR-Spo20^51–91-Sac1^2–517
Dtr1-mR-Sac1^2–517
WT
PD
WT
PD
5 μm

S2 Fig.

S2 Fig. Selective depletion of PI4P in the PSM suppresses the defects of *spo73*Δ.

Localization of $Sac1^{2–517}$ chimera proteins in *spo73*Δ (TC545) cells during PSM formation. mR, mRFP. PD, phosphatase-dead. Scale bar, 5 μm.
