# Supplementary material for: Suppression of Vps13 adaptor protein mutants reveals a central role for PI4P in regulating prospore membrane extension
Source: PLoS Genet. 2021 Aug 18;17(8):e1009727. doi: 10.1371/journal.pgen.1009727 (PMC8372973; doi:10.1371/journal.pgen.1009727)
Supplement: S3 Fig — (A) Localization of GFP-1×PHOsh2 (left) or GFP-2×PHOsh2 (right) in spo73Δ (TC545) cells overproducing mKate2-Spo2051–91-Sac12–517 during PSM formation. (B) Localization of GFP-P4M in wild-type cells producing Sec7-mRFP (TNY643) during vegetative growth. Sec7-mRFP, a Golgi marker. (C) Localization of GFP-P4M in wild-type (AN120) and spo73Δ (TC545) cells during PSM formation. mKate2-Spo2051–91, a PSM marker. (D) Localization of GFP-P4M in wild-type cells producing Sec7-mRFP and mTagBFP2-Spo2051–91 (TNY643) during PSM formation. Sec7, a Golgi marker. mTagBFP2-Spo2051–91, a PSM marker. mK, mKate2. Scale bar, 5 μm. (PDF) [file pgen.1009727.s003.pdf]

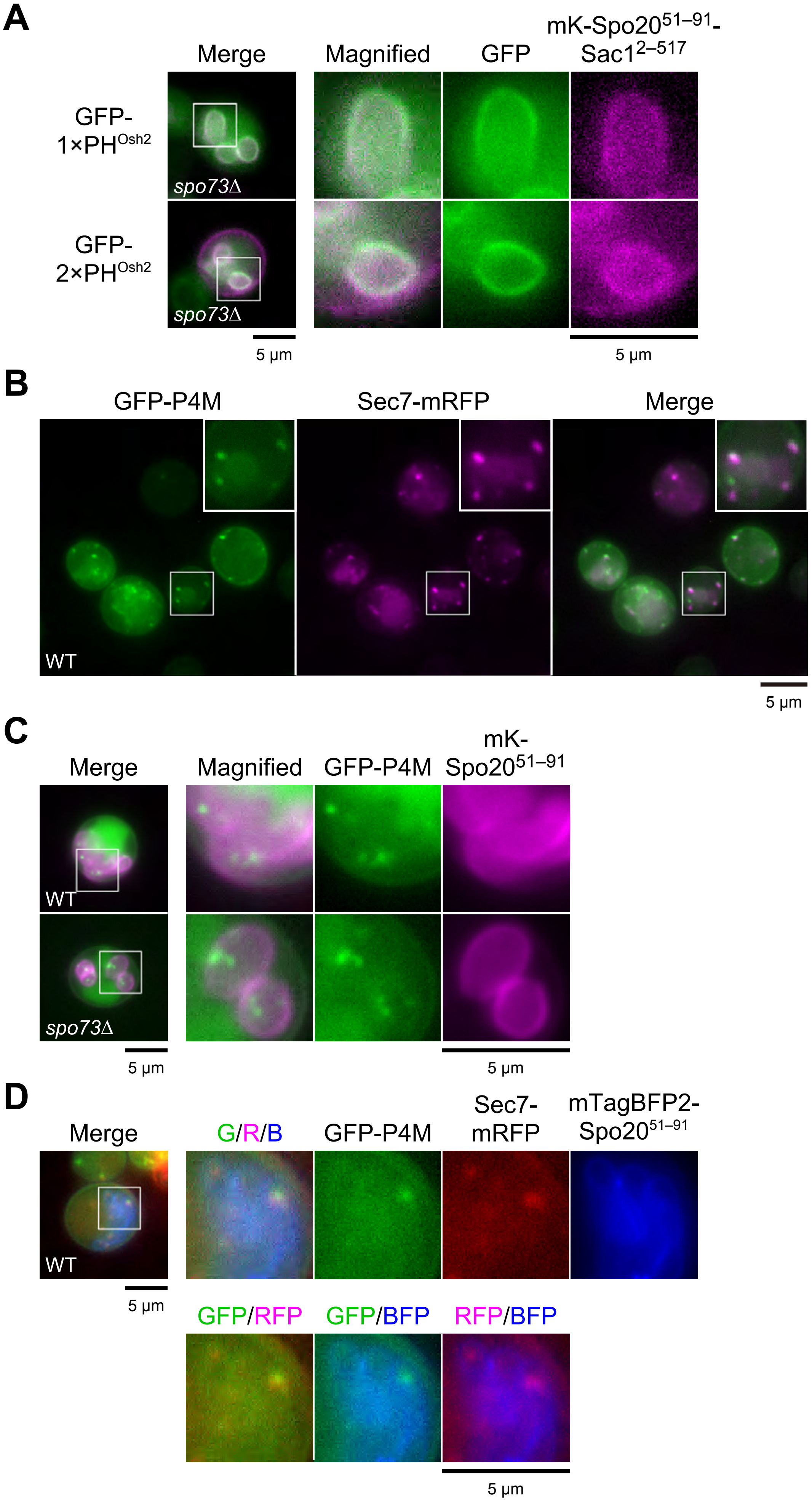
A
Merge
Magnified
GFP
mK-Spo20^51–91-Sac1^2–517
GFP-1×PH^Osh2
GFP-2×PH^Osh2
spo73Δ
spo73Δ
5 µm
5 µm
B
GFP-P4M
Sec7-mRFP
Merge
WT
5 µm
C
Merge
Magnified
GFP-P4M
mK-Spo20^51–91
WT
spo73Δ
5 µm
5 µm
D
Merge
G/R/B
GFP-P4M
Sec7-mRFP
mTagBFP2-Spo20^51–91
WT
5 µm
GFP/RFP
GFP/BFP
RFP/BFP
5 µm

S3 Fig.

S3 Fig. Changes in PI4P levels in the PSM can not be detected using conventional PI4P biomarkers.

(A) Localization of GFP-1×PH^{Osh2} (left) or GFP-2×PH^{Osh2} (right) in *spo73*Δ (TC545) cells overproducing mKate2-$Spo20^{51–91}$-$Sac1^{2–517}$ during PSM formation. (B) Localization of GFP-P4M in wild-type cells producing Sec7-mRFP (TNY643) during vegetative growth. Sec7-mRFP, a Golgi marker. (C) Localization of GFP-P4M in wild-type (AN120) and *spo73*Δ (TC545) cells during PSM formation. mKate2-$Spo20^{51–91}$, a PSM marker. (D) Localization of GFP-P4M in wild-type cells producing Sec7-mRFP and mTagBFP2-$Spo20^{51–91}$ (TNY643) during PSM formation. Sec7, a Golgi marker. mTagBFP2-$Spo20^{51–91}$, a PSM marker. mK, mKate2. Scale bar, 5 µm.
