# Supplementary material for: Suppression of Vps13 adaptor protein mutants reveals a central role for PI4P in regulating prospore membrane extension
Source: PLoS Genet. 2021 Aug 18;17(8):e1009727. doi: 10.1371/journal.pgen.1009727 (PMC8372973; doi:10.1371/journal.pgen.1009727)
Supplement: S4 Fig — (A and B) Localization of GFP-2×PHOsh2 (A), or GFP-Osh2-P4M (B) in wild-type (AN120) cells during vegetative growth. Two representative images are shown for each strain. (C) Localization of indicated PI4P markers in stt4-4 (AAY102) or pik1-83 (AAY104) cells during vegetative growth. These cells were observed after incubation at indicated temperatures for 60 min. (D) Localization of GFP-Osh2-P4M in wild-type cells expressing Sec7-mRFP and mTagBFP2-Spo2051–91 (TNY642) during PSM formation. Sec7-mRFP, a Golgi marker. mTagBFP2-Spo2051–91, a PSM marker. Scale bar, 5 μm. (PDF) [file pgen.1009727.s004.pdf]

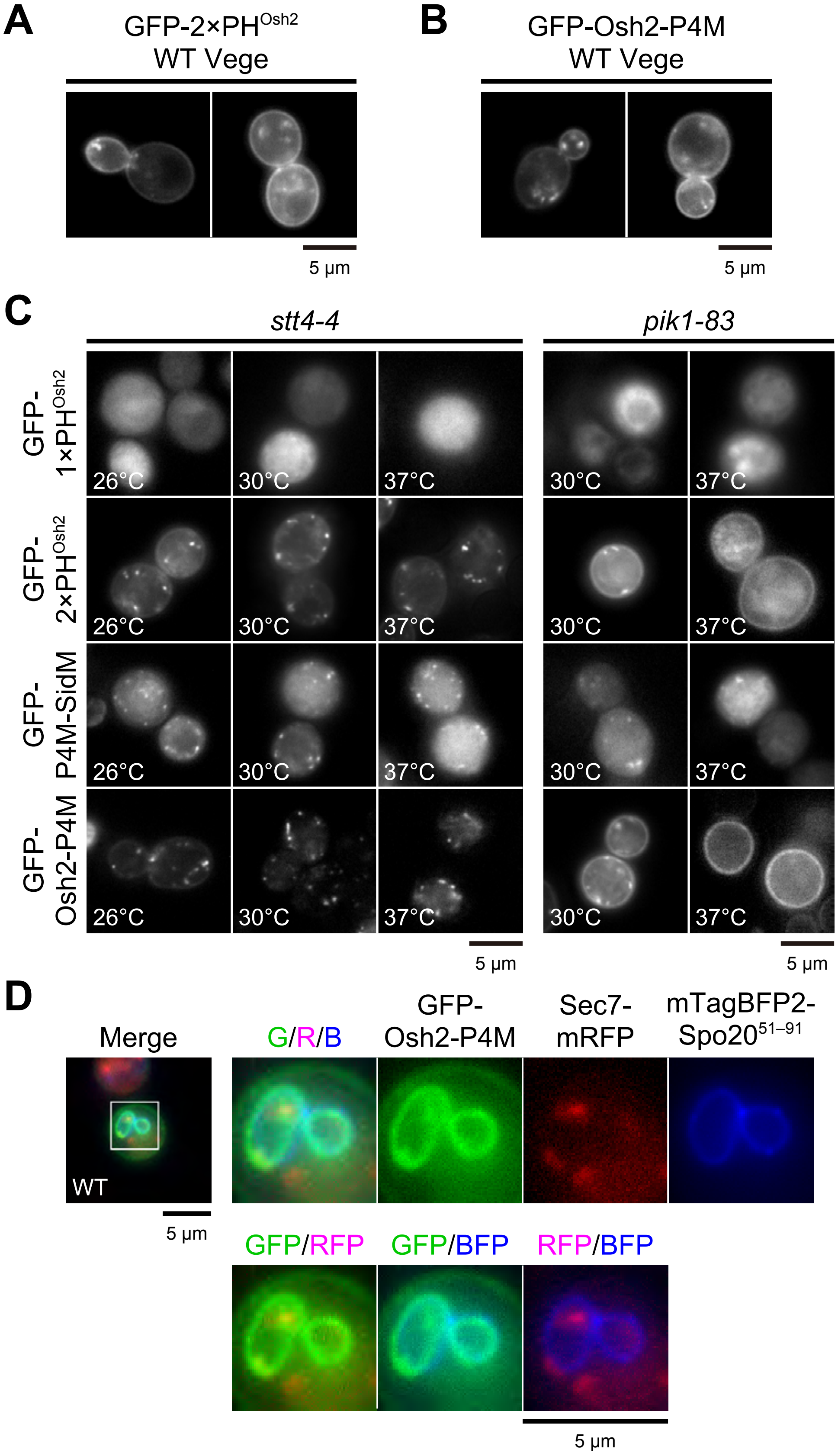

A
GFP-2×PH^{Osh2}
WT Vege
5 µm
B
GFP-Osh2-P4M
WT Vege
5 µm
C
stt4-4
pik1-83
GFP-1×PH^{Osh2}
GFP-2×PH^{Osh2}
GFP-P4M-SidM
GFP-Osh2-P4M
26°C
30°C
37°C
5 µm
D
Merge
WT
5 µm
G/R/B
GFP-Osh2-P4M
Sec7-mRFP
mTagBFP2-Spo20$^{51–91}$
GFP/RFP
GFP/BFP
RFP/BFP
5 µm

S4 Fig.

S4 Fig. Changes in PI4P levels in the PSM can be detected using an improved PI4P biomarker. (A and B) Localization of GFP-2×PH^{Osh2} (A), or GFP-Osh2-P4M (B) in wild-type (AN120) cells during vegetative growth. Two representative images are shown for each strain. (C) Localization of indicated PI4P markers in *stt4-4* (AAY102) or *pik1-83* (AAY104) cells during vegetative growth. These cells were observed after incubation at indicated temperatures for 60 min. (D) Localization of GFP-Osh2-P4M in wild-type cells expressing Sec7-mRFP and mTagBFP2-Spo20$^{51–91}$ (TNY642) during PSM formation. Sec7-mRFP, a Golgi marker. mTagBFP2-Spo20$^{51–91}$, a PSM marker. Scale bar, 5 μm.
