# Supplementary material for: Suppression of Vps13 adaptor protein mutants reveals a central role for PI4P in regulating prospore membrane extension
Source: PLoS Genet. 2021 Aug 18;17(8):e1009727. doi: 10.1371/journal.pgen.1009727 (PMC8372973; doi:10.1371/journal.pgen.1009727)
Supplement: S5 Fig — (A and B) Assessment of sporulation in spo73Δ (TC545, A) spo71Δ (TC581, B) overexpressing construct encoding mKate2-Spo2051–91-Sac12–517 and VPS13. More than 200 cells were observed in three independent colonies of each strain harboring indicated plasmids (for a total of > 600 cells). The bar graph shows mean ± SEM of the sporulation efficiency (N = 3). **, p < 0.01, ***, p < 0.001 (Tukey-Kramer test). (C) Ethanol resistance assay. spo71Δ (TC581) cells overexpressing VPS13 or construct encoding Spo71359–411-mKate2-Spo2051–91, or harboring empty vector, were sporulated for 2 days. Samples of 1 × 10^6^ cells of each transformant were treated with or without 26% ethanol (+EtOH or -EtOH) and inoculated onto YPD plates for 2 days. Magnification of inverted images of +EtOH YPD plates are shown. (D) Localization of overproduced Vps13^GFPEnvy in wild-type (AN120), spo73Δ (TC545) and spo71Δ (TC581) cells overexpressing construct encoding mKate2-Spo2051–91-Sac12–517 during PSM formation. mK, mKate2. Scale bar, 5 μm. (PDF) [file pgen.1009727.s005.pdf]

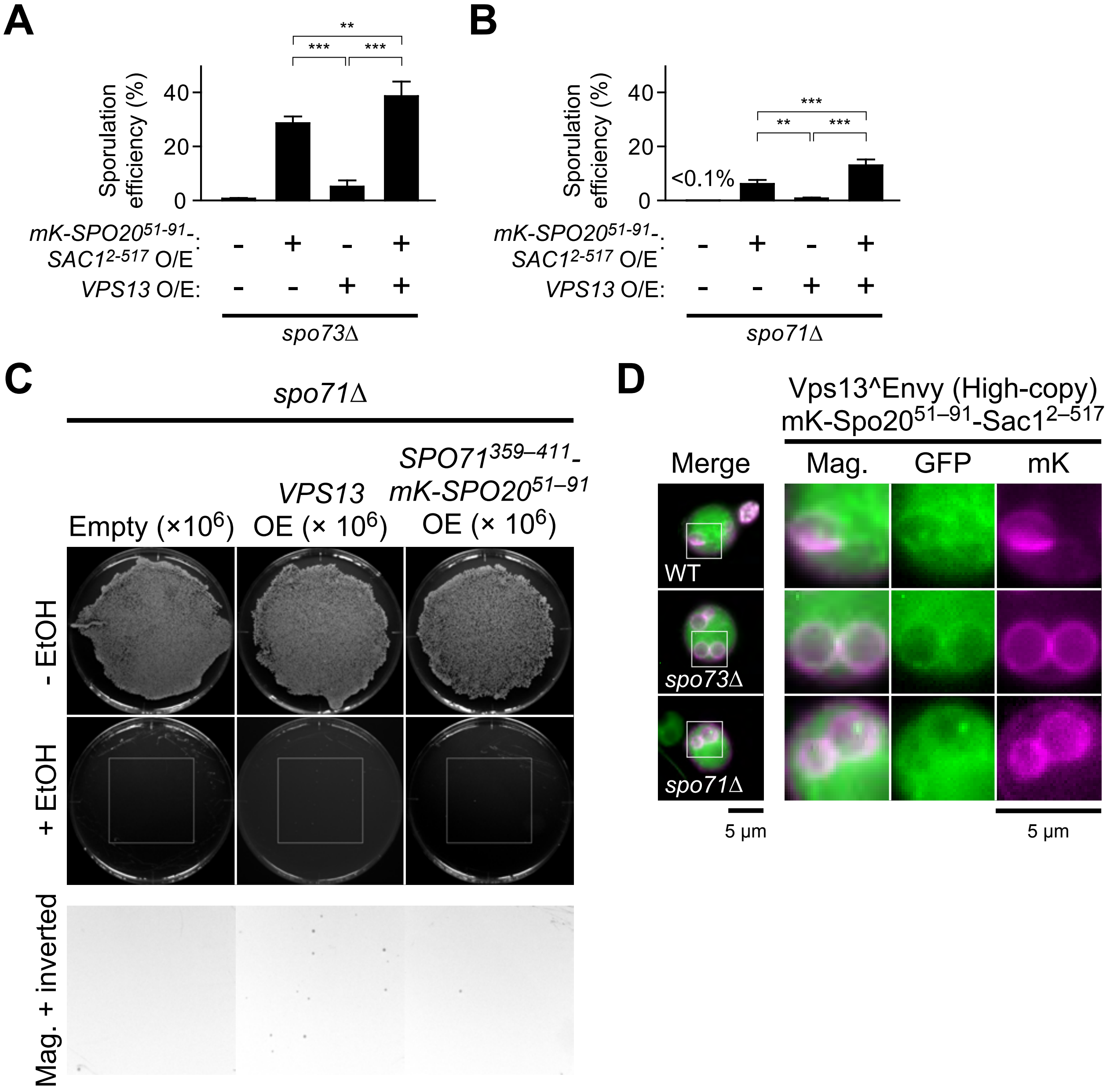
A
B
C
D
Sporulation efficiency (%)
40
20
0
<0.1%
**
***
mK-SPO20^51-91-SAC1^2-517 O/E:
VPS13 O/E:
spo73Δ
spo71Δ
Empty (×10^6)
VPS13 OE (× 10^6)
SPO71^359–411-mK-SPO20^51–91 OE (× 10^6)
- EtOH
+ EtOH
Mag. + inverted
Vps13^Envy (High-copy)
mK-Spo20^51–91-Sac1^2–517
Merge
Mag.
GFP
mK
WT
spo73Δ
spo71Δ
5 µm
5 µm

S5 Fig.

S5 Fig. Synergistic effects on suppression of Vps13 adaptor protein mutants.

(A and B) Assessment of sporulation in *spo73*Δ (TC545, A) *spo71*Δ (TC581, B) overexpressing construct encoding mKate2-Spo20$^{51–91}$-Sac1$^{2–517}$ and *VPS13*. More than 200 cells were observed in three independent colonies of each strain harboring indicated plasmids (for a total of > 600 cells). The bar graph shows mean ± SEM of the sporulation efficiency (N = 3). **, $p < 0.01$, ***, $p < 0.001$ (Tukey-Kramer test). (C) Ethanol resistance assay. *spo71*Δ (TC581) cells overexpressing *VPS13* or construct encoding Spo71$^{359–411}$-mKate2-Spo20$^{51–91}$, or harboring empty vector, were sporulated for 2 days. Samples of 1×10^6 cells of each transformant were treated with or without 26% ethanol (+EtOH or -EtOH) and inoculated onto YPD plates for 2 days. Magnification of inverted images of +EtOH YPD plates are shown. (D) Localization of overproduced Vps13^GFPEnvy in wild-type (AN120), *spo73*Δ (TC545) and *spo71*Δ (TC581) cells overexpressing construct encoding mKate2-Spo20$^{51–91}$-Sac1$^{2–517}$ during PSM formation. mK, mKate2. Scale bar, 5 µm.
