# Supplementary material for: Suppression of Vps13 adaptor protein mutants reveals a central role for PI4P in regulating prospore membrane extension
Source: PLoS Genet. 2021 Aug 18;17(8):e1009727. doi: 10.1371/journal.pgen.1009727 (PMC8372973; doi:10.1371/journal.pgen.1009727)
Supplement: S6 Fig — (A) Localization of indicated tethers for ER-PM contact sites in wild-type (TNY375, left) and spo73Δ (TNY376, right) cells during PSM formation. (B) Assessment of proximity of the PSM and the ER by BiFC assay. Wild-type (TNY375) cells producing mKate2-Spo2051–91-β1–10, Tcb3-GFP-β11, and mKate2-Spo2051–91 were observed during PSM formation. (C) Localization of Ist2 in spo71Δ (TNY544) and gip1Δ (TNY546) cells during PSM formation. Classification in inset; On the PSM: Class I and II, Not on the PSM: Class III. Line plot profiles of the white line in each panel. (D) Assessment of localization of Ist2 in indicated cells during PSM formation. Each strain expressing Ist2-GFP and mKate2-Spo2051–91 was observed during PSM formation, and PSM/peri-PSM ratios of the fluorescence of Ist2-GFP were calculated. More than 20 cells were measured in three independent colonies of each strain (for a total of 60 cells, respectively). The bar graph shows mean ± SEM of the percentage of cells (N = 3). *, p < 0.05, **, p < 0.01, ***, p < 0.001 (Tukey-Kramer test). (E) Assessment of localization of Ist2 in spo73Δ (TC611) and spo71Δ (TC609) cells overexpressing constructs encoding phosphatase active (WT) or phosphatase-dead (PD) Sac12–517 chimera proteins during PSM formation. More than 20 cells were measured in three independent colonies of each strain (for a total of 60 cells, respectively). The bar graph shows mean ± SEM of the percentage of cells (N = 3). n.s., not significant (Tukey-Kramer test). (F) Assessment of localization of Ist2 in spo71Δ (TC609) cells overexpressing constructs encoding Spo71359–411-mKate2-Spo2051–91 during PSM formation. More than 20 cells were measured in three independent colonies of each strain (for a total of 60 cells, respectively). The bar graph shows mean ± SEM of the percentage of cells (N = 3). n.s., not significant (Student’s t test). mK, mKate2. mKate2-Spo2051–91, a PSM marker. Scale bar, 5 μm. (PDF) [file pgen.1009727.s006.pdf]

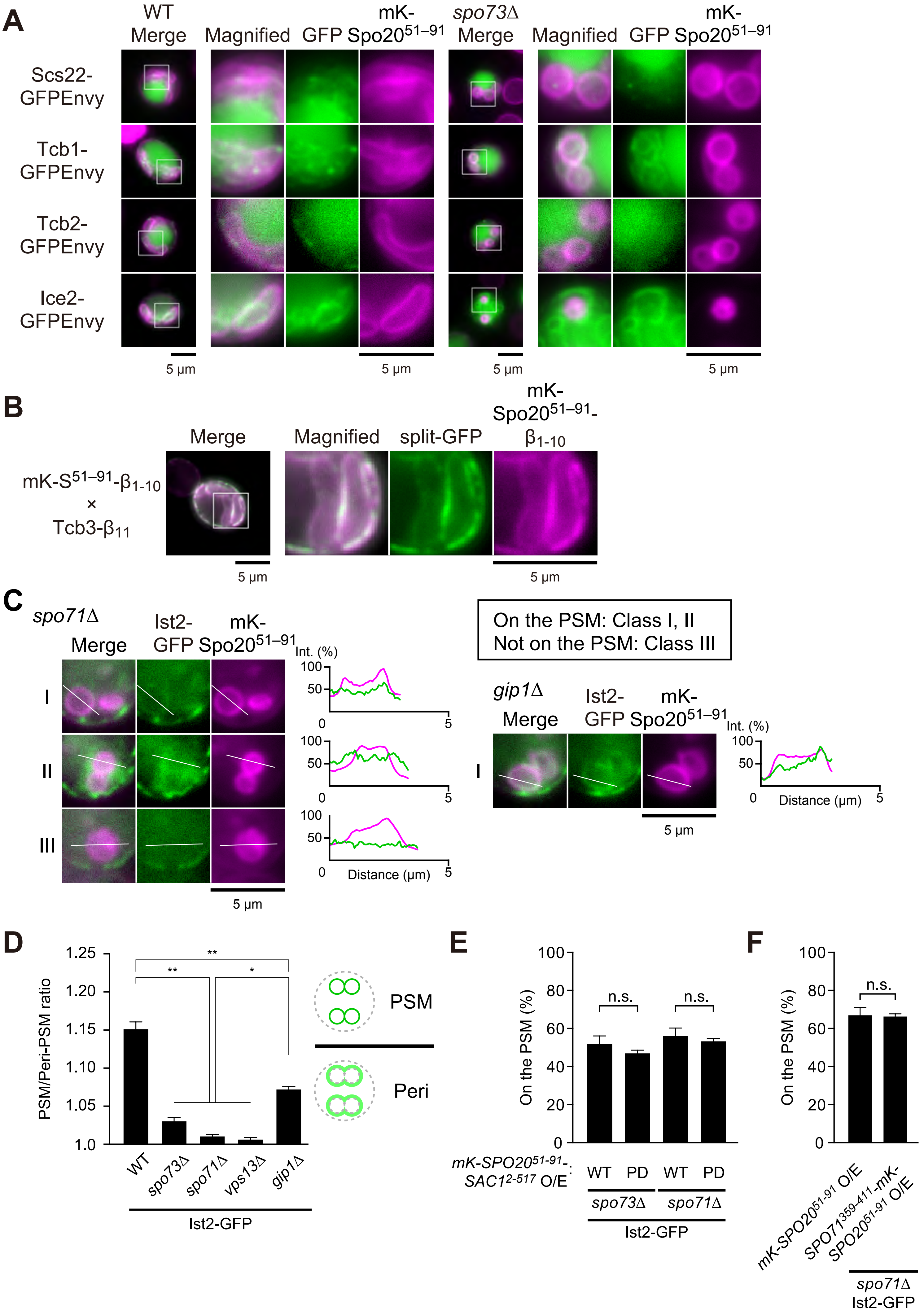
A
WT Merge
Magnified
GFP
mK-Spo20$^{51-91}$
spo73Δ Merge
Scs22-GFPEnvy
Tcb1-GFPEnvy
Tcb2-GFPEnvy
Ice2-GFPEnvy
5 μm
B
Merge
Magnified
split-GFP
mK-Spo20$^{51-91}$-β$_{1-10}$
mK-S$^{51-91}$-β$_{1-10}$ × Tcb3-β$_{11}$
5 μm
C
spo71Δ
Merge
Ist2-GFP
mK-Spo20$^{51-91}$
I
II
III
Int. (%)
100
50
0
5
Distance (μm)
5 μm
On the PSM: Class I, II
Not on the PSM: Class III
gip1Δ
D
PSM/Peri-PSM ratio
1.25
1.20
1.15
1.10
1.05
1.0
**
**
*
WT
spo73Δ
spo71Δ
vps13Δ
gip1Δ
Ist2-GFP
PSM
Peri
E
On the PSM (%)
100
80
60
40
20
0
n.s.
mK-SPO20$^{51-91}$-SAC1$^{2-517}$ O/E:
WT
PD
spo73Δ
spo71Δ
Ist2-GFP
F
mK-SPO20$^{51-91}$ O/E
SPO71$^{359-411}$-mK-SPO20$^{51-91}$ O/E
spo71Δ Ist2-GFP

S6 Fig.

S6 Fig. ER-PM tethers localize along the PSM dependently on Vps13 and adaptor proteins. (A) Localization of indicated tethers for ER-PM contact sites in wild-type (TNY375, left) and *spo73*Δ (TNY376, right) cells during PSM formation. (B) Assessment of proximity of the PSM and the ER by BiFC assay. Wild-type (TNY375) cells producing mKate2-Spo20$^{51–91}$-$\beta_{1\text{-}10}$, Tcb3-GFP-β11, and mKate2-Spo20$^{51–91}$ were observed during PSM formation. (C) Localization of Ist2 in *spo71*Δ (TNY544) and *gip1*Δ (TNY546) cells during PSM formation. Classification in inset; On the PSM: Class I and II, Not on the PSM: Class III. Line plot profiles of the white line in each panel. (D) Assessment of localization of Ist2 in indicated cells during PSM formation. Each strain expressing Ist2-GFP and mKate2-Spo20$^{51–91}$ was observed during PSM formation, and PSM/peri-PSM ratios of the fluorescence of Ist2-GFP were calculated. More than 20 cells were measured in three independent colonies of each strain (for a total of 60 cells, respectively). The bar graph shows mean ± SEM of the percentage of cells (N = 3). *, $p < 0.05$, **, $p < 0.01$, ***, $p < 0.001$ (Tukey-Kramer test). (E) Assessment of localization of Ist2 in *spo73*Δ (TC611) and *spo71*Δ (TC609) cells overexpressing constructs encoding phosphatase active (WT) or phosphatase-dead (PD) Sac1$^{2–517}$ chimera proteins during PSM formation. More than 20 cells were measured in three independent colonies of each strain (for a total of 60 cells, respectively). The bar graph shows mean ± SEM of the percentage of cells (N = 3). n.s., not significant (Tukey-Kramer test). (F) Assessment of localization of Ist2 in *spo71*Δ (TC609) cells overexpressing constructs encoding Spo71$^{359–411}$-mKate2-Spo20$^{51–91}$ during PSM formation. More than 20 cells were measured in three independent colonies of each strain (for a total of 60 cells, respectively). The bar graph shows mean ± SEM of the percentage of cells (N = 3). n.s., not significant (Student's t test). mK, mKate2. mKate2-Spo20$^{51–91}$, a PSM marker. Scale bar, 5 μm.
