# Supplementary material for: Suppression of Vps13 adaptor protein mutants reveals a central role for PI4P in regulating prospore membrane extension
Source: PLoS Genet. 2021 Aug 18;17(8):e1009727. doi: 10.1371/journal.pgen.1009727 (PMC8372973; doi:10.1371/journal.pgen.1009727)
Supplement: S7 Fig — Localization of indicated Osh proteins in wild-type (TNY375) cells during PSM formation. mKate2-Spo2051–91, a PSM marker. Scale bar, 5 μm. (PDF) [file pgen.1009727.s007.pdf]

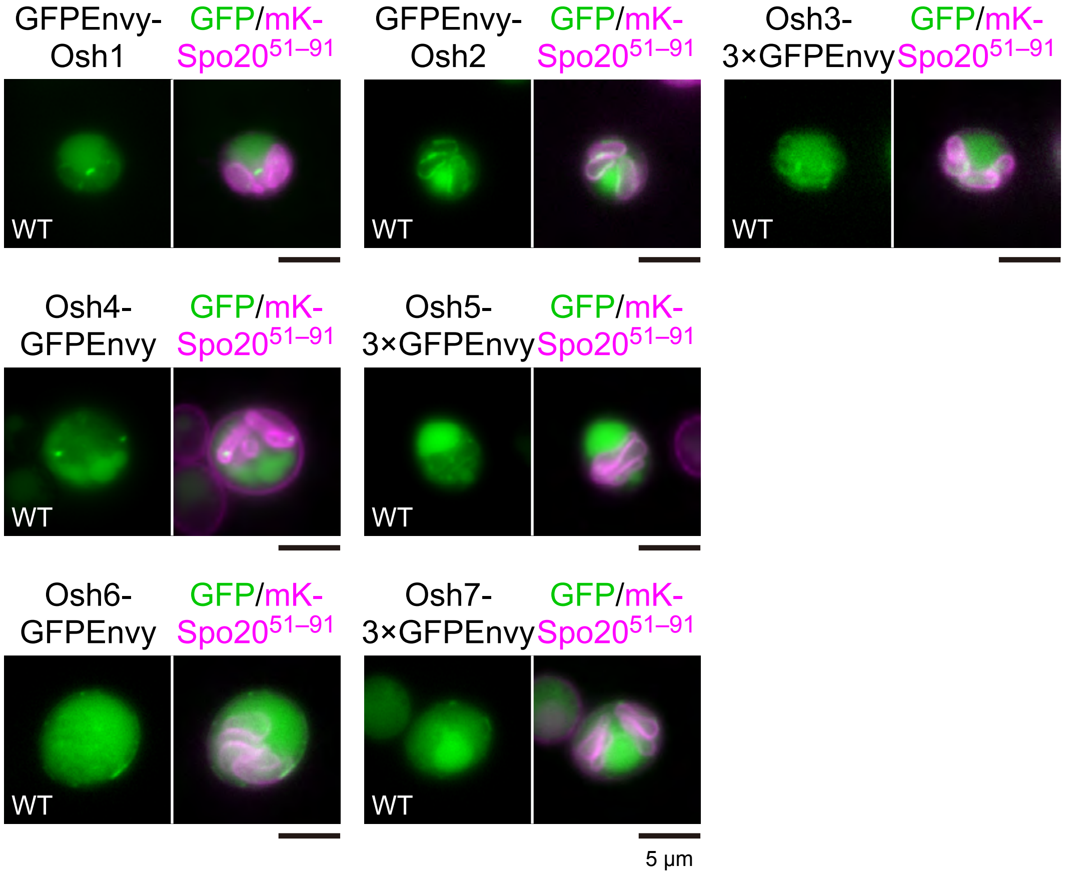
GFPEnvy-Osh1
GFP/mK-Spo20$^{51–91}$
GFPEnvy-Osh2
GFP/mK-Spo20$^{51–91}$
Osh3-3×GFPEnvy
GFP/mK-Spo20$^{51–91}$
Osh4-GFPEnvy
GFP/mK-Spo20$^{51–91}$
Osh5-3×GFPEnvy
GFP/mK-Spo20$^{51–91}$
Osh6-GFPEnvy
GFP/mK-Spo20$^{51–91}$
Osh7-3×GFPEnvy
GFP/mK-Spo20$^{51–91}$
WT
WT
WT
WT
WT
WT
WT
5 μm

S7 Fig.

S7 Fig. Localization of Osh proteins during PSM formation.

Localization of indicated Osh proteins in wild-type (TNY375) cells during PSM formation. mKate2-Spo20$^{51–91}$, a PSM marker. Scale bar, 5 μm.
