# Supplementary material for: Suppression of Vps13 adaptor protein mutants reveals a central role for PI4P in regulating prospore membrane extension
Source: PLoS Genet. 2021 Aug 18;17(8):e1009727. doi: 10.1371/journal.pgen.1009727 (PMC8372973; doi:10.1371/journal.pgen.1009727)
Supplement: S1 Table — (PDF) [file pgen.1009727.s013.pdf]

S1 Table. Strains used for this study.

Strain	Description	Genotype	Source	Figure
AN120	Wild-type	MATa/MATα his3 ΔSK/his3 ΔSK ura3/ura3 trp1::hisG/trp1::hisG leu2/leu2<br>arg4-NspI/ARG4 lys2/lys2 ho Δ::LYS2/ho Δ::LYS2 rme1::LEU2/RME1	Ref. 1	Figure 1
TC545	spo73 Δ	AN120 spo73::kanMX6/spo73::kanMX6	Ref. 2	Figure 1
AH109	Strain for yeast two-hybrid assay	MATa trp1-901 leu2-3, 112 ura3-52 his3-200 gal4 Δ gal80 Δ LYS2::GAL1 $_{UAS}$-GAL1 $_{TATA}$-HIS3<br>GAL2 $_{UAS}$-GAL2 $_{TATA}$-ADE2 URA3::MEL1 $_{UAS}$-MEL1 $_{TATA}$-lacZ	Clontech	Figure 1
AAY102	stt4-4	SEY6210 (MATα leu2-3, 112 ura3-52 his3- Δ200 trp1- Δ901 lys2-801 suc2- Δ9)<br>stt4::HIS3MX6 carrying pRS415-stt4-4 (LEU2 CEN6 stt4-4)	Ref. 3	Sup. Figure 1
TNY168	Wild-type NDT80-B/R	AN120 AUR1:: P_{ACT1}-LexA-ER-haVP16::AUR1-C/AUR1:: P_{ACT1}-LexA-ER-haVP16::AUR1-C<br>ndt80::hphNT1:: $P_{4\times lexA}$-9×Myc-NDT80/ndt80::hphNT1:: $P_{4\times lexA}$-9×Myc-NDT80	This study	Figure 3
TNY150	spo73 Δ NDT80-B/R	TNY168 spo73::kanMX6/spo73::kanMX6	This study	Figure 3
TNY502	spo73 Δ NDT80-B/R P_{CLB2}-degron-stt4	TNY168 spo73::kanMX6/spo73::kanMX6 his3 ΔSK:: P_{ADH1}-mod-OsTIR1::HIS3/his3 ΔSK:: P_{ADH1}-mod-OsTIR1::HIS3<br>stt4::natNT2:: P_{CLB2}-degron-stt4/stt4::natNT2:: P_{CLB2}-degron-stt4	This study	Figure 3
TNY517	spo73 Δ NDT80-B/R P_{CLB2}-degron-stt4<br>P_{ADH1}-GFP-STT4	TNY502 ura3:: P_{ADH1}-GFP-STT4::URA3/ura3	This study	Figure 3
TNY198	spo73 Δ NDT80-B/R mKate2-SPO20 $^{51-91}$	TNY150 his3 ΔSK:: P_{TEF1}-mKate2-SPO20 $^{51-91}$::HIS3/his3 ΔSK	This study	Figure 3
TNY518	spo73 Δ NDT80-B/R P_{CLB2}-degron-stt4<br>mKate2-SPO20 $^{51-91}$	TNY502 P_{TEF1}-mKate2-SPO20 $^{51-91}$::URA3/ura3	This study	Figure 3
TNY578	spo73 Δ NDT80-B/R<br>$P_{4\times lexA}$-3×mK-SPO20 $^{51-91}$-SAC1 $^{2-517}$	TNY150 ura3:: $P_{4\times lexA}$-3×mK-SPO20 $^{51-91}$-SAC1 $^{2-517}$::URA3/ura3	This study	Figure 4
TNY643	Wild-type<br>P_{TDH3}-GFP-P4M-SidM SEC7-mRFP	AN120 ura3:: P_{TDH3}-GFP-P4M-SidM::URA3/ura3 trp1::hisG:: P_{ADH1}-SEC7-mRFP/trp1::hisG	This study	Sup. Figure 3
AAY104	pik1-83	SEY6210 (MATα leu2-3, 112 ura3-52 his3- Δ200 trp1- Δ901 lys2-801 suc2- Δ9)<br>pik-83::HIS3MX6 carrying pRS314-pik1-83 (LEU2 CEN6 pik1-83)	Ref. 3	Sup. Figure 4
TNY642	Wild-type<br>P_{TDH3}-GFP-OSH2-P4M SEC7-mRFP	AN120 ura3:: P_{TDH3}-GFP-OSH2-P4M::URA3/ura3 trp1::hisG:: P_{ADH1}-SEC7-mRFP/trp1::hisG	This study	Sup. Figure 4
TNY375	Wild-type mKate2-SPO20 $^{51-91}$	AN120 his3 ΔSK:: P_{TEF1}-mKate2-SPO20 $^{51-91}$::HIS3/his3 ΔSK	This study	Figure 5
TNY376	spo73 Δ mKate2-SPO20 $^{51-91}$	TC545 his3 ΔSK:: P_{TEF1}-mKate2-SPO20 $^{51-91}$::HIS3/his3 ΔSK	This study	Figure 5
TNY403	spo73 Δ NDT80-B/R<br>P_{TDH3}-GFP-OSH2-P4M mKate2-SPO20 $^{51-91}$	TNY150 his3 ΔSK:: P_{TDH3}-GFP-OSH2-P4M::HIS3/his3 ΔSK:: P_{TEF1}-mKate2-SPO20 $^{51-91}$::HIS3	This study	Figure 5
TNY421	spo73 Δ NDT80-B/R<br>P_{TDH3}-GFP-OSH2-P4M<br>$P_{4\times lexA}$-3×mK-SPO20 $^{51-91}$-SAC1 $^{2-517}$	TNY150 his3 ΔSK:: P_{TDH3}-GFP-OSH2-P4M::HIS3/his3 ΔSK ura3:: $P_{4\times lexA}$-3×mK-SPO20 $^{51-91}$-SAC1 $^{2-517}$::URA3/ura3	This study	Figure 5
TNY429	spo73 Δ NDT80-B/R<br>P_{TDH3}-GFP-OSH2-P4M<br>$P_{4\times lexA}$-3×mK-SPO20 $^{51-91}$-SAC1 $^{2-517}$-PD	TNY150 his3 ΔSK:: P_{TDH3}-GFP-OSH2-P4M::HIS3/his3 ΔSK ura3:: $P_{4\times lexA}$-3×mK-SPO20 $^{51-91}$-SAC1 $^{2-517}$-PD::URA3/ura3	This study	Figure 5
TNY473	Wild-type NDT80-B/R P_{CLB2}-degron-stt4	TNY168 his3 ΔSK:: P_{ADH1}-mod-OsTIR1::HIS3/his3 ΔSK:: P_{ADH1}-mod-OsTIR1::HIS3<br>stt4::natNT2:: P_{CLB2}-degron-stt4/stt4::natNT2:: P_{CLB2}-degron-stt4	This study	Figure 6

S1 Table. Strains used for this study (continued).

Strain	Description	Genotype	Source	Figure
TNY519	Wild-type *NDT80* -B/R P_{CLB2} *-degron-stt4* $P_{4\times lexA}$ *-3×mK-SPO20* $^{51-91}$ *-SAC1* $^{2-517}$	TNY473 *ura3::* $P_{4\times lexA}$ *-3×mK-SPO20* $^{51-91}$ *-SAC1* $^{2-517}$ *::URA3/ura3*	This study	Figure 6
TNY197	Wild-type *NDT80* -B/R *mKate2-SPO20* $^{51-91}$	TNY168 *his3* Δ*SK::* P_{TEF1} *-mKate2-SPO20* $^{51-91}$ *::HIS3* /*his3* Δ*SK*	This study	Figure 6
TC581	*spo71* Δ	AN120 *spo71::kanMX6* /*spo71::kanMX6*	Ref. 2	Figure 7
TNY637	*spo71* Δ *spo73* Δ	AN120 *spo71::natNT2* /*spo71::natNT2 spo73::kanMX6* /*spo73::kanMX6*	This study	Figure 7
TC572	*vps13* Δ	AN120 *vps13::kanMX6* /*vps13::kanMX6*	Ref. 2	Figure 7
AN117-4B	Wild-type (*MAT* α)	*MAT* α *his3* Δ*SK ura3 trp1::hisG leu2 arg4-NspI lys2 ho* Δ*::LYS2 rme1::LEU2*	Ref. 1	-
AN117-16Da	Wild-type (*MAT* a)	*MAT* a *his3* Δ*SK ura3 trp1::hisG leu2 lys2 ho* Δ*::LYS2*	Ref. 1	-
TNY475	*VPS13^TRP1* (*MAT* α)	AN117-4B *VPS13::TRP1* $_{1360}$	This study	-
YFY30	*VPS13^GFPEnvy-loxP-HIS3MX6-loxP* (*MAT* α)	AN117-4B *VPS13::GFPEnvy* $_{1360}$ *-loxP-HIS3MX6-loxP*	This study	-
YFY29	*VPS13^GFPEnvy-loxP-HIS3MX6-loxP* (*MAT* a)	AN117-16Da *VPS13::GFPEnvy* $_{1360}$ *-loxP-HIS3MX6-loxP*	This study	-
YFY49	*VPS13^GFPEnvy* (*MAT* α)	AN117-4B *VPS13::GFPEnvy* $_{1360}$	This study	-
YFY39	*VPS13^GFPEnvy* (*MAT* a)	AN117-16Da *VPS13::GFPEnvy* $_{1360}$	This study	-
YFY57	Wild-type *VPS13^GFPEnvy*	AN120 *VPS13::GFPEnvy* $_{1360}$ /*VPS13::GFPEnvy* $_{1360}$	This study	Figure 7
YFY83	Wild-type *VPS13^GFPEnvy mKate2-SPO20* $^{51-91}$	YFY57 *his3* Δ*SK::* P_{TEF1} *-mKate2-SPO20* $^{51-91}$ *::HIS3* /*his3* Δ*SK*	This study	Figure 7
YFY85	*spo73* Δ *VPS13^GFPEnvy mKate2-SPO20* $^{51-91}$	YFY83 *spo73::kanMX6/spo73::kanMX6*	This study	Figure 7
YFY84	*spo71* Δ *VPS13^GFPEnvy mKate2-SPO20* $^{51-91}$	YFY83 *spo71::natNT2/spo71::natNT2*	This study	Figure 7
YFY59	*spo71* Δ *VPS13^GFPEnvy*	AN120 *VPS13::GFPEnvy* $_{1360}$ /*VPS13::GFPEnvy* $_{1360}$ *spo71::natNT2/spo71::natNT2*	This study	Figure 7
TC544	*gip1* Δ	AN120 *gip1::kanMX6* /*gip1::kanMX6*	Ref. 4	Figure 8
TNY522	Wild-type *IST2-GFP mKate2-SPO20* $^{51-91}$	AN120 *his3* Δ*SK::* P_{TEF1} *-mKate2-SPO20* $^{51-91}$ *::HIS3* /*his3* Δ*SK ura3::IST2-GFP::URA3/ura3*	This study	Figure 8
TNY524	*spo73* Δ *IST2-GFP mKate2-SPO20* $^{51-91}$	TNY522 *spo73::kanMX6/spo73::kanMX6*	This study	Figure 8
TNY544	*spo71* Δ *IST2-GFP mKate2-SPO20* $^{51-91}$	TNY522 *spo71::kanMX6/spo71::kanMX6*	This study	Figure 8
TNY545	*vps13* Δ *IST2-GFP mKate2-SPO20* $^{51-91}$	TNY522 *vps13::kanMX6/vps13::kanMX6*	This study	Figure 8
TNY546	*gip1* Δ *IST2-GFP mKate2-SPO20* $^{51-91}$	TNY522 *gip1::kanMX6/gip1::kanMX6*	This study	Figure 8
TNY659	Wild-type *TCB2-GFP mKate2-SPO20* $^{51-91}$	AN120 *his3* Δ*SK::* P_{TEF1} *-mKate2-SPO20* $^{51-91}$ *::HIS3* /*his3* Δ*SK TCB2::GFPEnvy::HIS3MX6/TCB2*	This study	Sup. Figure 6
TNY660	*spo73* Δ *TCB2-GFP mKate2-SPO20* $^{51-91}$	TNY522 *spo73::kanMX6/spo73::kanMX6*	This study	Sup. Figure 6
TC611	*spo73* Δ *IST2-GFP*	TC545 *ura3::IST2-GFP::URA3/ura3*	This study	Sup. Figure 6
TC609	*spo71* Δ *IST2-GFP*	TC581 *ura3::IST2-GFP::URA3/ura3*	This study	Sup. Figure 6

Reference

1. Neiman AM, Katz L, Brennwald PJ. Identification of domains required for developmentally regulated SNARE function in *Saccharomyces cerevisiae* . Genetics. 2000;155: 1643–1655.
2. Okumura Y, Nakamura TS, Tanaka T, Inoue I, Suda Y, Takahashi T, et al. The dysferlin domain-only protein, Spo73, is required for prospore membrane extension in *Saccharomyces cerevisiae* . Mitchell AP, editor. mSphere. 2016;1: e00038-15. doi:10.1128/mSphere.00038-15
3. Audhya A, Emr SD. Stt4 PI 4-kinase localizes to the plasma membrane and functions in the Pkc1-mediated MAP kinase cascade. Dev Cell. 2002;2: 593–605. doi:10.1016/S1534-5807(02)00168-5
4. Nakamura TS, Numajiri Y, Okumura Y, Hidaka J, Tanaka T, Inoue I, et al. Dynamic localization of a yeast development-specific PP1 complex during prospore membrane formation is dependent on multiple localization signals and complex formation. Mol Biol Cell. 2017;28: 3881–3895. doi:10.1091/mbc.E17-08-0521
