# Supplementary material for: Suppression of Vps13 adaptor protein mutants reveals a central role for PI4P in regulating prospore membrane extension
Source: PLoS Genet. 2021 Aug 18;17(8):e1009727. doi: 10.1371/journal.pgen.1009727 (PMC8372973; doi:10.1371/journal.pgen.1009727)
Supplement: S2 Table — (PDF) [file pgen.1009727.s014.pdf]

S2 Table. Plasmids used for this study.

Number	Name	Protein Expressed/Description	Source	Used Primer	PCR template	Used Plasmid	Figure
TNP1311	pRS424	High-copy vector	Ref. 1	-	-	-	Figure 1 A
TNP1401	pRS424-T_{CYC1}	T_{CYC1}	Ref. 2	-	-	-	-
TNP0219	pRS424-STT4frag	Stt4frag O/P	This study	TN42, TN64	gDNA of AN120	pRS424-T_{CYC1}	Figure 1 A
TNP0101	pRS424-EFR3	Efr3 O/P	This study	TN66, TN67	gDNA of AN120	pRS424	Figure 1 A
TNP1402	pRS424-P_{TEF1}	P_{TEF1}	Ref. 2	-	-	-	-
TNP1428	pRS424-P_{TEF1}-C-GFP-T_{ADH1}	P_{TEF1}-C-GFP-T_{ADH1}	Ref. 2	-	-	-	-
TNP0110	pRS424-P_{TEF1}-EFR3-1–2346bp-GFP	Efr3-1–782 aa-GFP O/P	This study	TN49, TN70	gDNA of AN120	pRS424-P_{TEF1}-C-GFP-T_{ADH1}	Figure 1 C
TNP0114	pRS424-P_{TEF1}-EFR3-1–2190bp-GFP	Efr3-1–730 aa-GFP O/P	This study	TN49, TN219	gDNA of AN120	pRS424-P_{TEF1}-C-GFP-T_{ADH1}	Figure 1 C
TNP0113	pRS424-P_{TEF1}-EFR3-1–1950bp-GFP	Efr3-1–650 aa-GFP O/P	This study	TN49, TN217	gDNA of AN120	pRS424-P_{TEF1}-C-GFP-T_{ADH1}	Figure 1 C
TNP0117	pRS424-P_{TEF1}-EFR3-1951–2346bp-GFP	Efr3-651–782 aa-GFP O/P	This study	TN50, TN246	gDNA of AN120	pRS424-P_{TEF1}-C-GFP-T_{ADH1}	Figure 1 C
TNP1404	pRS424-P_{TEF1}-T_{CYC1}	P_{TEF1}-T_{CYC1}	This study	-	-<br>-	pRS424-T_{CYC1}<br>pRS424-P_{TEF1}	-
TNP0221	pRS424-P_{TEF1}-STT4-1–2442bp	Stt4-1–814 aa O/P	This study	TN41, TN42	gDNA of AN120	pRS424-P_{TEF1}-T_{CYC1}	Figure 1 D
TNP0225	pRS424-P_{TEF1}-STT4-1–1494bp	Stt4-1–498 aa O/P	This study	TN41, TN82	gDNA of AN120	pRS424-P_{TEF1}-T_{CYC1}	Figure 1 D
TNP0222	pRS424-P_{TEF1}-STT4-1–1221bp	Stt4-1–407 aa O/P	This study	TN41, TN47	gDNA of AN120	pRS424-P_{TEF1}-T_{CYC1}	Figure 1 D
TNP0224	pRS424-P_{TEF1}-STT4-1–771bp	Stt4-1–257 aa O/P	This study	TN41, TN79	gDNA of AN120	pRS424-P_{TEF1}-T_{CYC1}	Figure 1 D
TNP0227	pRS424-P_{TEF1}-STT4-1–522bp	Stt4-1–184 aa O/P	This study	TN41, TN105	gDNA of AN120	pRS424-P_{TEF1}-T_{CYC1}	Figure 1 D
TNP0228	pRS424-P_{TEF1}-STT4-1–405bp	Stt4-1–135 aa O/P	This study	TN41, TN106	gDNA of AN120	pRS424-P_{TEF1}-T_{CYC1}	Figure 1 D
TNP0233	pRS424-P_{TEF1}-STT4-406–2442bp	Stt4-136–814 aa O/P	This study	TN42, TN116	gDNA of AN120	pRS424-P_{TEF1}-T_{CYC1}	Figure 1 D
TNP0864	pGADT7	Yeast two-hybrid system vector	Clontech	-	-	-	-
TNP0867	pGBKT7	Yeast two-hybrid system vector	Clontech	-	-	-	-
TNP0875	pBG4D-2	Yeast two-hybrid system vector	Gift from T. Maeda	-	-	-	Figure 1 E
TNP0251	pGBKT7-EFR3-1951–2346bp	GBD-Efr3-651–782 aa	This study	TN49, TN70	gDNA of AN120	pGBKT7	-
TNP0262	pBG4D-2-EFR3-1951–2346bp	Efr3-651–782 aa-GBD	This study	-	-	pGBKT7-EFR3-1951–2346bp<br>pBG4D-2	Figure 1 E
TNP0255	pBG4D-2-STT4-1–2442bp	Stt4-1–814 aa-GBD	This study	TN41, TN76	gDNA of AN120	pBG4D-2-EFR3-1951–2346bp	Figure 1 E
TNP0257	pBG4D-2-STT4-1–771bp	Stt4-1–257 aa-GBD	This study	TN41, TN78	gDNA of AN120	pBG4D-2-EFR3-1951–2346bp	Figure 1 E
TNP0258	pBG4D-2-STT4-1–552bp	Stt4-1–184 aa-GBD	This study	TN41, TN338	gDNA of AN120	pBG4D-2-EFR3-1951–2346bp	Figure 1 E
TNP0259	pBG4D-2-STT4-1–405bp	Stt4-1–135 aa-GBD	This study	TN41, TN379	gDNA of AN120	pBG4D-2-EFR3-1951–2346bp	Figure 1 E
TNP0260	pBG4D-2-STT4-406–2442bp	Stt4-136–814 aa-GBD	This study	TN76, TN116	gDNA of AN120	pBG4D-2-EFR3-1951–2346bp	Figure 1 E
TNP0263	pGADT7-YPP1	GAD-Ypp1	This study	TN145, TN146	gDNA of AN120	pGADT7	Figure 1 E
TNP1308	pRS316	Low-copy vector	Ref. 3	-	-	-	-
TNP1328	pRS314-P_{ADH1}-GFP-Nterm	P_{ADH1}-N-GFP-T_{CYC1}	Ref. 4	-	-	-	-
TNP1329	pRS316-P_{ADH1}-GFP-Nterm	P_{ADH1}-N-GFP-T_{CYC1}	This study	-	-	pRS314-P_{ADH1}-GFP-Nterm<br>pRS316	-
TNP0211	pRS316-P_{ADH1}-GFP-STT4	GFP-Stt4 low-copy	This study	YSMO024, YMSO025	gDNA of YPH499	pRS316-P_{ADH1}-GFP-Nterm	Figure 2 A
TNP0214	pRS424-P_{ADH1}-GFP-STT4	GFP-Stt4 high-copy	This study	-	-	pRS316-P_{ADH1}-GFP-STT4<br>pRS424	Figure 2 A
TNP1222	pRS426-P_{TEF1}-mRFP-SPO20$^{51-91}$ (pRS426-R20)	mRFP-Spo20$^{51-91}$	Ref. 5	-	-	-	Figure 2 A
TNP1036	pFA6a-GFP(S65T)-kanMX6	C-terminal tagging GFP-kanMX6	Ref. 6	-	-	-	-
TNP1331	pRS316-P_{ADH1}-GFP-Cterm	P_{ADH1}-C-GFP-T_{ADH1}	This study	YSMO012, YSMO013<br>YSMO017, YSMO018	gDNA of YPH499<br>pFA6a-GFP(S65T)-kanMX6	pRS316	-
TNP0105	pRS316-EFR3-GFP	Efr3-GFP low-copy	This study	YSMO028, YSMO029	gDNA of YPH499	pRS316-P_{ADH1}-GFP-Cterm	Figure 2 C
TNP0106	pRS424-EFR3-GFP	Efr3-GFP high-copy	This study	-	-	pRS316-EFR3-GFP<br>pRS424	Figure 2 C

S2 Table. Plasmids used for this study (continued).

Number	Name	Protein Expressed/Description	Source	Used Primer	PCR template	Used Plasmid	Figure
TNP1241	pRS316-P_{TEF1}-mKate2-SPO20$^{51–91}$	mKate2-Spo20$^{51–91}$	Ref. 2	-	-	-	Figure 2 E
TNP1038	pFA6a-mCherry-HIS3MX6	C-terminal tagging mCherry-HIS3MX6	Gift from M. Onishi	-	-	-	-
TNP1312	pRS426	High-copy vector	Ref. 1	-	-	-	-
TNP1429	pRS424-P_{TEF1}-C-mCherry-T_{ADH1}	P_{TEF1}-C-mCherry-T_{ADH1}	This study	TN98. TN99	pFA6a-mCherry-HIS3MX6	pRS424-P_{TEF1}-C-GFP-T_{ADH1}	-
TNP0123	pRS424-P_{TEF1}-EFR3-mCherry	Efr3-mCherry O/P	This study	-	-	pRS424-P_{TEF1}-EFR3-1–2346bp-GFP<br>pRS424-P_{TEF1}-C-mCherry-T_{ADH1}	-
TNP0124	pRS426-P_{TEF1}-EFR3-mCherry	Efr3-mCherry O/P	This study	-	-	pRS424-P_{TEF1}-EFR3-mCherry<br>pRS426	Figure 2 E
TNP1307	pRS314	Low-copy vector	Ref. 3	-	-	-	-
TNP0201	pRS314-STT4	Stt4	This study	TN57, TN64	gDNA of AN120	pRS314	Sup. Fig. 1 A
TNP0202	pRS314-STT4-D1754A	Stt4^{D1754A}	This study	TN121, TN122	pRS314-STT4	-	Sup. Fig. 1 A
TNP0203	pRS424-STT4	Stt4 O/P	This study	-	-	pRS314-STT4, pRS424	Sup. Fig. 1 B
TNP0204	pRS424-STT4-D1754A	Stt4^{D1754A} O/P	This study	-	-	pRS314-STT4-D1754A, pRS424	Sup. Fig. 1 B
TNP1033	pFA6a-link-GFPEnvy-SpHis5	C-terminal tagging GFPEnvy-SpHis5	Addgene (#60782)	-	-	-	-
TNP1338	pRS314-P_{TEF1}-T_{CYC1}	P_{TEF1}-T_{CYC1}	This study	-	-	pRS314<br>pRS424-P_{TEF1}-T_{CYC1}	-
TNP1355	pRS314-P_{TEF1}-N-GFPEnvy-T_{CYC1}	P_{TEF1}-N-GFPEnvy-T_{CYC1}	This study	TN396, TN397	pFA6a-GFPEnvy-HIS3MX6	pRS314-P_{TEF1}-T_{CYC1}	-
TNP0179	pRS314-P_{TEF1}-GFPEnvy-YPP1	GFPEnvy-Ypp1	This study	TN145. TN146	gDNA of AN120	pRS314-P_{TEF1}-N-GFPEnvy-T_{CYC1}	Sup. Fig. 1 C
TNP1346	pRS314-C-GFP-T_{ADH1}	C-GFP-T_{ADH1}	This study	-	-	pRS314<br>pRS424-P_{TEF1}-C-GFP-T_{ADH1}	-
TNP0172	pRS314-SFK1-GFP	Sfk1-GFP	This study	TN163, TN390	gDNA of AN120	pRS314-C-GFP-T_{ADH1}	Sup. Fig. 1 D
TNP1061	pAUR101	Integration to *AUR1* locus	Takara	-	-	-	-
TNP1055	FRP467	Original P_{ACT1} *-LexA-ER-VP16*	Addgene (#58430)	-	-	-	-
TNP1056	mod_FRP467	StuI site in *ER* is omitted.	This study	TN289, TN290	FRP467	-	-
TNP1059	pAUR101-P_{ACT1}-LexA-ER-VP16	modified P_{ACT1} *-LexA-ER-VP16* (*AUR1-C*)	This study	-	-	mod_FRP467 (#58430)<br>pAUR101	Figure 3 B
TNP1047	pRK69	P_{CLB} *-N-3×HA* tag (*kanMX6*)	Ref. 7	-	-	-	-
TNP1001	pFA6a-HIS3MX6	Template of *HIS3MX6*	Ref. 6	-	-	-	-
TNP1046	pFA6a-HIS3MX6-P_{CLB2}-3×HA	P_{CLB2} *-N-3×HA* tag (*HIS3MX6*)	This study	-	-	pRK69<br>pFA6a-HIS3MX6	-
TNP1110	pMK38	Template of P_{CUP1} *-N-IAA17-linker*	NRBP (#BYP6739)	-	-	-	-
TNP1111	pFA6a-HIS3MX6-P_{CLB2}-IAA17	P_{CLB2} *-N-N-IAA17-linker* tag (*HIS3MX6*)	This study	TN187, TN188	pMK38	pFA6a-HIS3MX6-P_{CLB2}-3×HA	-
TNP1128	pMK27	P_{GAL1} *-AtTIR1-9×Myc::URA3*	NRBP (#BYP6741)	-	-	-	-
TNP1052	pFA6a-kanMX6-P_{CLB2}-9×Myc	P_{CLB2} *-N-9×Myc-linker* tag (*kanMX6*)	This study	HJ47, HJ48	pMK27	pRK69<br>pFA6a-HIS3MX6-P_{CLB2}-IAA17	-
TNP1124	pMK152	*C-degron* tag (*hphNT1*)	NBRP (#BYP7431)	-	-	-	-
TNP1064	FRP1642	$P_{4×lexA}$ tag (*hphNT1*)	Addgene (#58442)	-	-	-	-
TNP1065	pFA6a-hphNT1-$P_{4×lexA}$-9×Myc	$P_{4×lexA}$ *-9×Myc-linker* tag (*hphNT1*)	This study	TN310, TN311	FBP1642	pFA6a-kanMX6-P_{CLB2}-9×Myc<br>pMK152	Figure 3 B
TNP1048	pFA6a-natNT2-P_{CLB2}-3HA	P_{CLB2} *-N-3HA* tag (*natNT2*)	Ref. 4	-	-	-	-
TNP1119	pFA6a-natNT2-P_{CLB2}-degron	P_{CLB2} *-N-degron-linker tag* (*natNT2*)	This study	TN205, TN206	pMK152	pFA6a-natNT2-P_{CLB2}-3HA<br>pFA6a-kanMX6-P_{CLB2}-9×Myc	Figure 3 B
TNP1140	pMK200	P_{ADH1} *-mod-OsTIR1:: URA3*	NBRP (#BYP7569)	-	-	-	-
TNP1301	pRS303	Integration vector	Ref. 3	-	-	-	-

S2 Table. Plasmids used for this study (continued).

Number	Name	Protein Expressed/Description	Source	Used Primer	PCR template	Used Plasmid	Figure
TNP1139	pRS303-P_{ADH1}-mod-OsTIR1	P_{ADH1} *-mod-OsTIR1:: HIS3*	This study	-	-	pMK200<br>pRS303	Figure 3 B
TNP1303	pRS306	Integration vector	Ref. 3	-	-	-	-
TNP0210	pRS306-P_{ADH1}-GFP-STT4	P_{ADH1} *-GFP-STT4::URA3*	This study	-	-	pRS316-P_{ADH1}-GFP-STT4<br>pRS306	Figure 3 D
TNP1310	mod_pRS303	KpnI site in *HIS3* is omitted.	Ref. 2	-	-	-	-
TNP1237	mod_pRS303-P_{TEF1}-<br>mKate2-SPO20$^{51–91}$	mKate2-Spo20$^{51–91}$	This study	-	-	mod_pRS303<br>pRS316-P_{TEF1}-mKate2-SPO20$^{51–91}$	Figure 3 E
TNP0313	pRS424-SAC1	Sac1 O/P	This study	TN119, TN120	gDNA of AN120	pRS424	Figure 4 B
TNP1408	pRS424-P_{SPR3}-T_{CYC1}	P_{SPR3}-T_{CYC1}	Ref. 2	-	-	-	-
TNP0679	pRS424-P_{SPR3}-mRFP-<br>SPO20$^{51–91}$-[SAGG]×4- T_{CYC1}	P_{SPR3}-mRFP-SPO20$^{51–91}$-[SAGG]×4- T_{CYC1}	This study	TN95, TN100	pRS424-P_{TEF1}-mRFP-SPO20$^{51–91}$	pRS424-P_{SPR3}-T_{CYC1}	-
TNP0335	pRS424-P_{SPR3}-mR-SPO20$^{51–91}$-SAC1$^{2–517}$	mR-Spo20$^{51–91}$-Sac1$^{2–517}$ O/P	This study	TN101, TN102	gDNA of AN120	pRS424-P_{SPR3}-mRFP-<br>SPO20$^{51–91}$-[SAGG]×4- T_{CYC1}	Figure 4 B
TNP0337	pRS424-P_{SPR3}-mR-SPO20$^{51–91}$-SAC1$^{2–517}$-PD	mR-Spo20$^{51–91}$-Sac1$^{2–517}$-PD O/P	This study	TN143, TN144	pRS424-P_{SPR3}-mR-SPO20$^{51–91}$-SAC1$^{2–517}$	-	Figure 4 B
TNP0475	pRS424-DTR1-mRFP	Dtr1-mRFP	Ref. 8	-	-	-	-
TNP0680	pRS424-DTR1-mRFP-[SAGG]×4- T_{CYC1}	DTR1-mRFP-[SAGG]×4- T_{CYC1}	This study	TN127, TN128	pRS424-DTR1-mRFP	pRS424-T_{CYC1}	-
TNP0355	pRS424-DTR1-mR-SAC1$^{2–517}$	Dtr1-mR-Sac1$^{2–517}$ O/P	This study	TN101, TN102	gDNA of AN120	pRS424-DTR1-mRFP-[SAGG]×4- T_{CYC1}	Figure 4 B
TNP0357	pRS424-DTR1-mR-SAC1$^{2–517}$-PD	Dtr1-mR-Sac1$^{2–517}$-PD O/P	This study	TN143, TN144	pRS424-DTR1-mR-SAC1$^{2–517}$	-	Figure 4 B
TNP1337	pRS314-T_{CYC1}	T_{CYC1}	This study	-	-	pRS314<br>pRS424-T_{CYC1}	-
TNP1067	pRS314-$P_{4×lexA}$-T_{CYC1}	$P_{4×lexA}$-T_{CYC1}	This study	pRS-F, TN386	FRP1642	pRS314-T_{CYC1}	-
TNP1068	pRS306-$P_{4×lexA}$-T_{CYC1}	$P_{4×lexA}$ -T_{CYC1}*::URA3*	This study	-	-	pRS314-$P_{4×lexA}$-T_{CYC1}<br>pRS306	-
TNP1366	pRS314-P_{TEF1}-N-3×mKate2-T_{CYC1}	P_{TEF1}-N-3×mKate2-T_{CYC1}	This study	TN357, TN358<br>TN421, TN423	pRS316-P_{TEF1}-mKate2-SPO20$^{51–91}$	pRS314-P_{TEF1}-T_{CYC1}	-
TNP0353	pRS314-$P_{4×lexA}$-3×mK-SPO20$^{51–91}$-SAC1$^{2–517}$	$P_{4×lexA}$-3×mK-Spo20$^{51–91}$-Sac1$^{2–517}$	This study	-	-	pRS424-P_{SPR3}-mR-SPO20$^{51–91}$-SAC1$^{2–517}$<br>pRS314-P_{TEF1}-N-3×mKate2-T_{CYC1}<br>pRS314-$P_{4×lexA}$-T_{CYC1}	-
TNP0351	pRS306-$P_{4×lexA}$-3×mK-SPO20$^{51–91}$-SAC1$^{2–517}$	$P_{4×lexA}$ *-3×mK-SPO20* $^{51–91}$ *-SAC1* $^{2–517}$ *::URA3*	This study	-	-	pRS314-$P_{4×lexA}$-3×mK-SPO20$^{51–91}$-SAC1$^{2–517}$<br>pRS306-$P_{4×lexA}$-T_{CYC1}	Figure 4 C
TNP1513	pRS416-P_{PHO5}-GFP-2×PH^{OSH2}-GFP (pTL511)	GFP-2×PH^{Osh2}	Ref. 9	-	-	-	Sup. Fig. 3 A
TNP1502	pRS416-P_{PHO5}-GFP-1×PH^{OSH2}-GFP	GFP-1×PH^{Osh2}	This study	-	-	pRS416-P_{PHO5}-GFP-2×PH^{OSH2}-GFP	Sup. Fig. 3 A
TNP0333	pRS424-P_{SPR3}-mK-SPO20$^{51–91}$-SAC1$^{2–517}$	mK-Spo20$^{51–91}$-Sac1$^{2–517}$ O/P	This study	-	-	pRS316-P_{TEF1}-mKate2-SPO20$^{51–91}$<br>pRS424-P_{SPR3}-mR-SPO20$^{51–91}$-SAC1$^{2–517}$	Sup. Fig. 3 A
TNP1530	pRS426-P_{PRC1}-GFP-FYVE^{EEA1}	GFP-FYVE^{EEA1} O/P	Gift from T. Noda	-	-	-	-
TNP1340	pRS314-P_{TDH3}-T_{CYC1}	P_{TDH3}-T_{CYC1}	This study	TN279, TN280	gDNA of AN120	pRS314-T_{CYC1}	-
-	pRS416	Low-copy vector	Ref. 3	-	-	-	-
TNP1529	pRS416-P_{TDH3}-GFP-FYVE^{EEA1}	GFP-FYVE^{EEA1}	This study	TN27, pRS-F	pRS426-P_{PRC1}-GFP-FYVE^{EEA1}	pRS314-P_{TDH3}-T_{CYC1}<br>pRS416-P_{PHO5}-GFP-1×PH^{OSH2}-GFP	Figure 6 B
TNP1538	mCherry-P4M-SidM	mCherry-P4M-SidM	Addgene (#51471)	-	-	-	-
TNP1541	pRS416-P_{TDH3}-GFP-P4M-SidM	GFP-P4M-SidM	This study	-	-	mCherry-P4M-SidM<br>pRS416-P_{TDH3}-GFP-FYVE^{EEA1}	Sup. Fig. 3 C

S2 Table. Plasmids used for this study (continued).

Number	Name	Protein Expressed/Description	Source	Used Primer	PCR template	Used Plasmid	Figure
TNP1539	pRS306-P_{TDH3}-GFP-P4M-SidM	*GFP-P4M::URA3*	This study	-	-	pRS306<br>pRS416-P_{TDH3}-GFP-P4M-SidM	Sup. Fig. 3 B
TNP1302	pRS304	Integration vector	Ref. 3	-	-	-	-
TNP1624	pRS316-P_{ADH1}-SEC7-mRFP	Sec7-mRFP	Ref. 10	-	-	-	-
TNP1623	pRS314-P_{ADH1}-SEC7-mRFP	Sec7-mRFP	This study	-	-	pRS316-P_{ADH1}-SEC7-mRFP<br>pRS314	-
TNP1621	pRS304-P_{ADH1}-SEC7-mRFP	P_{ADH1} -*SEC7-mRFP::TRP1*	This study	-	-	pRS304<br>pRS314-P_{ADH1}-SEC7-mRFP	-
TNP1622	pRS304mod_P_{ADH1}-SEC7-mRFP	P_{ADH1} -*SEC7-mRFP::TRP1*	This study	-	-	pRS304-P_{ADH1}-SEC7-mRFP	Sup. Fig. 3 B
TNP1240	pRS314-P_{TEF1}-mKate2-SPO20$^{51–91}$	mKate2-Spo20$^{51–91}$	This study	-	-	pRS316-P_{TEF1}-mKate2-SPO20$^{51–91}$<br>pRS314	Sup. Fig. 3 C
TNP1305	pRS313	Low-copy vector	Ref. 3	-	-	-	-
TNP1306	mod_pRS313	Modified low-copy vector	This study	-	-	FRP467<br>pRS313	-
TNP1239	mod_pRS313-P_{TEF1}-<br>mKate2-SPO20$^{51–91}$	mKate2-Spo20$^{51–91}$	This study	-	-	pRS316-P_{TEF1}-mKate2-SPO20$^{51–91}$<br>mod_pRS313	-
TNP1257	mod_pRS313-P_{TEF1}-<br>mTagBFP2-SPO20$^{51–91}$	mTagBFP2-Spo20$^{51–91}$	This study	TN408, TN409	*mTagBFP2* -gBlocks	mod_pRS303-P_{TEF1}-<br>mKate2-SPO20$^{51–91}$	Sup. Fig. 3 D
TNP1537	GFP-P4M-SidM	GFP-P4M-SidM	Addgene (#51469)	-	-	-	-
TNP1549	pRS424-P_{TEF1}-GFP-OSH2-P4M	GFP-Osh2-P4M	This study	-	-	pRS416-P_{PHO5}-GFP-2×PH^{OSH2}-GFP<br>GFP-P4M-SidM<br>pRS424-P_{TEF1}	-
TNP1548	pRS416-P_{TDH3}-GFP-OSH2-P4M	GFP-Osh2-P4M	This study	TN27, pRS-F	pRS424-P_{TEF1}-GFP-OSH2-P4M	pRS416-P_{TDH3}-GFP-FYVE^{EEA1}	Figure 5 B
TNP1304	mod_pRS306	SmaI site is created in *URA3* .	This study	TN542, TN543	pRS306	-	-
TNP1547	mod_pRS306-P_{TDH3}-GFP-P4M-SidM	*GFP-OSH2-P4M::URA3*	This study	-	-	mod_pRS303-P_{TDH3}-GFP-P4M-SidM<br>mod_pRS306	Sup. Fig. 4 D
TNP1546	mod_pRS303-P_{TDH3}-GFP-OSH2-P4M	*GFP-OSH2-P4M::HIS3*	This study	-	-	pRS416-P_{TDH3}-GFP-P4M-SidM<br>mod_pRS303	Figure 5 C
TNP0352	pRS306-$P_{4×lexA}$-3×mK-SPO20$^{51–91}$-SAC1$^{2–517}$-PD	$P_{4×lexA}$-*3×mK-SPO20* $^{51–91}$ -*SAC1* $^{2–517}$ -*PD::URA3*	This study	-	-	pRS424-P_{SPR3}-mR-SPO20$^{51–91}$-SAC1$^{2–517}$-PD<br>pRS306-$P_{4×lexA}$-3×mK-SPO20$^{51–91}$-SAC1$^{2–517}$<br>pRS306-$P_{4×lexA}$-T_{CYC1}	Figure 5 C
TNP1523	pRS426-P_{PRC1}-GFP-2×PH^{PLCδ}	GFP-2×PH^{PLCδ} O/P	Ref. 11	-	-	-	-
TNP1524	pRS416-P_{TDH3}-GFP-2×PH^{PLCδ}	GFP-2×PH^{PLCδ}	This study	-	-	pRS426-P_{PRC1}-GFP-2×PH^{PLCδ}<br>pRS416-P_{TDH3}-GFP-FYVE^{EEA1}	Figure 6 C
TNP1360	pRS314-P_{TEF1}-N-3×GFPEnvy-T_{CYC1}	P_{TEF1}-N-3×GFPEnvy-T_{CYC1}	This study	TN394, TN396<br>TN397, TN414<br>TN422	pRS314-P_{TEF1}-N-GFPEnvy-T_{CYC1}	pRS314-P_{TEF1}-T_{CYC1}	-
TNP0140	pRS314-P_{MSS4}-3×GFPEnvy-MSS4	3×GFPEnvy-Mss4	This study	TN123, TN550<br>TN126, TN393	gDNA of AN120	pRS314-P_{TEF1}-N-3×GFPEnvy<br>-T_{CYC1}	Figure 6 D
TNP1004	pFA6a-kanMX6	Template of *kanMX6*	Ref. 6	-	-	-	-
TNP1920	pFA6a-spo71Δ-kanMX6-Long	Template of *spo71::kanMX6*	This study	TN804, TN805	TC581 genome	pFA6a-kanMX6	-
TNP1005	pFA6a-natNT2	Template of *natNT2*	Ref. 12	-	-	pFA6a-natNT2-P_{CLB2}-3HA	-
TNP1923	pFA6a-spo71Δ-natNT2-Long	Template of *spo71::natNT2*	This study	-	-	pFA6a-natNT2<br>pFA6a-spo71Δ-kanMX6-Long	Figure 7 A
TNP0449	pRS426-VPS13	Vps13 O/P	Ref. 13	-	-	-	Figure 7 B

S2 Table. Plasmids used for this study (continued).

Number	Name	Protein Expressed/Description	Source	Used Primer	PCR template	Used Plasmid	Figure
TNP1002	pFA6a-TRP1	Template of *TRP1*	Ref. 6	-	-	-	-
TNPP110	pRS424-VPS13-TRP1(1360)-frag	Frangemnt of *VPS13* containing *TRP1* insertion	This study	TN635, TN636	TNY475 genome	pRS424	-
TNPP109	pRS424-VPS13-GFPEnvy(1360)-frag	Frangemnt of *VPS13* containing *GFPEnvy* insertior	This study	-	-	pRS314-P$_{TEF1}$-N-GFPEnvy-T$_{CYC1}$<br>pRS424-VPS13-TRP1(1360)-frag	-
TNP0448	pRS314-VPS13	Vps13	This study	-	-	pRS426-VPS13<br>pRS314	-
TNP0532	pRS314-VPS13-GFPEnvy(1360)	Vps13^GFPEnvy	This study	TN701, TN702<br>TN633, TN634<br>TN703, TN704	gDNA of AN120<br>pRS424-VPS13-GFPEnvy(1360)-frag	pRS314-VPS13	-
TNP1930	pFA6a-VPS13-GFPEnvy(1360)-frag-Long	Frangemnt of *VPS13* containing *GFPEnvy* insertior	This study	TN801, TN802	pRS314-VPS13-GFPEnvy(1360)	pFA6a-kanMX6	-
TNP1014	pFA6a-loxP-hphNT1-loxP (pUG-hph)	Template of *loxP-hphNT1-loxP*	Ref. 14	-	-	-	-
TNP1016	pFA6a-loxP-HIS3MX6-loxP (pUG-His)	Template of *loxP-HIS3MX6-loxP*	This study	-	-	pFA6a-HIS3MX6<br>pUG-hph	-
TNP1931	pFA6a-VPS13-GFPEnvy(1360)-loxP-HIS3MX6-loxP-Long	Frangemnt of *VPS13*<br>containing GFPEnvy::loxP-HIS3MX6-loxP insertion	This study	TN807, TN810	pUG-His	pFA6a-VPS13-GFPEnvy(1360)-frag-Long	Figure 7 C
TNP1955	pRS425-Cas9-SkHIS3-381	Cas9 and gRNA for *SkHIS3-381*	Ref. 15	-	-	-	-
TNP1956	pRS426-Cas9-SkHIS3-381	Cas9 and gRNA for *SkHIS3-381*	This study	YF17, YF18	pRS425-Cas9-SkHIS3-381	pRS426	Figure 7 C
TNP0437	pRS426-SPO71	Spo71 O/P	Ref. 13	-	-	-	-
TNP0436	pRS314-SPO71	Spo71	This study	-	-	pRS426-SPO71<br>pRS314	Figure 7 C
TNP1946	pRS314-P$_{TEF1}$-C-mKate2-SPO20$^{51–91}$-T$_{ADH1}$	P$_{TEF1}$-C-mKate2-Spo20$^{51–91}$-T$_{ADH}$	This study	YF12, YF13	pRS316-P$_{TEF1}$-mKate2-SPO20$^{51–91}$	pRS314-P$_{TEF1}$-C-GFPEnvy-T$_{ADH1}$	-
TNP1947	pRS314-P$_{TEF1}$-SPO71$^{359–411}$-mK-SPO20$^{51–91}$	Spo71$^{359–411}$-mK-Spo20$^{51–91}$	This study	YF9, YF10	pRS314-SPO71	pRS314-P$_{TEF1}$-C-mKate2-SPO20$^{51–91}$-T$_{ADH1}$	-
TNP1957	pRS424-P$_{SPR3}$-SPO71$^{359–411}$-mK-SPO20$^{51–91}$	Spo71$^{359–411}$-mK-Spo20$^{51–91}$ O/P	This study	-	-	pRS314-P$_{TEF1}$-SPO71$^{359–411}$-mK-SPO20$^{51–91}$<br>pRS424-P$_{SPR3}$-T$_{CYC1}$	Figure 7 D
TNP1958	pRS426-P$_{SPR3}$-SPO71$^{359–411}$-mK-SPO20$^{51–91}$	Spo71$^{359–411}$-mK-Spo20$^{51–91}$ O/P	This study	-	-	pRS424-P$_{SPR3}$-SPO71$^{359–411}$-mK-SPO20$^{51-91}$<br>pRS426	Figure 7 F
TNP1954	pRS314-P$_{TEF1}$-VPS13$^{1–57}$-GFPEnvy-T$_{ADH1}$	Vps13$^{1–57}$-GFPEnvy	This study	YF3, YF63	pRS314-VPS13-GFPEnvy(1360)	pRS314-P$_{TEF1}$-C-GFPEnvy-T$_{ADH1}$	Figure 7 G
TNP1373	pRS316-P$_{TEF1}$-T$_{CYC1}$	P$_{TEF1}$-T$_{CYC1}$	This study	-	-<br>-	pRS424-P$_{TEF1}$-T$_{CYC1}$<br>pRS316	-
TNP1377	pRS316-P$_{TEF1}$-N-mKate2-T$_{CYC1}$	P$_{TEF1}$-N-mKate2-T$_{CYC1}$	This study	TN357, TN358	pRS316-P$_{TEF1}$-mKate2-SPO20$^{51–91}$	pRS316-P$_{TEF1}$-T$_{CYC1}$	-
TNP1857	pRS316-P$_{TEF1}$-mKate2-SEC61-T$_{CYC1}$	mKate2-Sec61	This study	TN466, TN467	gDNA of AN120	pRS316-P$_{TEF1}$-N-mKate2-T$_{CYC1}$	Figure 7 G
TNP0533	pRS426-VPS13-GFPEnvy(1360)	Vps13^GFPEnvy O/P	This study	-	-	pRS314-VPS13-GFPEnvy(1360)<br>pRS426-VPS13	Sup. Fig. 5 D
TNP1829	pRS314-IST2-GFP	Ist2-GFP	This study	TN317, TN318	gDNA of AN120	pRS314-C-GFP-T$_{ADH1}$	Figure 8 A
TNP1034	pFA6a-GFPEnvy-HIS3MX6	C-terminal tagging GFPEnvy-HIS3MX6	This study	-	-	pFA6a-HIS3MX6<br>pFA6a-link-GFPEnvy-SpHis5	-
TNP1350	pRS314-P$_{TEF1}$-C-GFP-T$_{ADH1}$	P$_{TEF1}$-C-GFP-T$_{ADH1}$	Ref. 2	-	-	-	-
TNP1358	pRS314-P$_{TEF1}$-C-GFPEnvy-T$_{ADH1}$	P$_{TEF1}$-C-GFPEnvy-T$_{ADH1}$	This study	TN394, TN395	pFA6a-GFPEnvy-HIS3MX6	pRS314-P$_{TEF1}$-C-GFP-T$_{ADH1}$	-
TNP1840	pRS314-P$_{TEF1}$-TCB3-GFPEnvy	Tcb3-GFPEnvy	This study	TN500, TN501	gDNA of AN120	pRS314-P$_{TEF1}$-C-GFPEnvy-T$_{ADH1}$	Figure 8 A
TNP1833	pRS314-SCS2-GFPEnvy	Scs2-GFPEnvy	This study	TN490, TN491	gDNA of AN120	pRS314-P$_{TEF1}$-C-GFPEnvy-T$_{ADH1}$	Figure 8 A
TNP1828	pRS306-IST2-GFP	*IST2-GFP::URA3*	This study	-	-	pRS314-IST2-GFP<br>pRS306	Figure 8 B
TNP1835	pRS314-P$_{TEF1}$-SCS22-GFPEnvy	Scs22-GFPEnvy	This study	TN821, TN822	gDNA of AN120	pRS314-P$_{TEF1}$-C-GFPEnvy-T$_{ADH1}$	Sup. Fig. 6 A
TNP1837	pRS314-P$_{TEF1}$-TCB1-GFPEnvy	Tcb1-GFPEnvy	This study	TN817, TN818	gDNA of AN120	pRS314-P$_{TEF1}$-C-GFPEnvy-T$_{ADH1}$	Sup. Fig. 6 A
TNP1841	pRS314-P$_{TEF1}$-ICE2-GFPEnvy	Ice2-GFPEnvy	This study	TN823, TN824	gDNA of AN120	pRS314-P$_{TEF1}$-C-GFPEnvy-T$_{ADH1}$	Sup. Fig. 6 A
TNP1375	pRS316-P$_{TEF1}$-C-GFPEnvy-T$_{ADH1}$	C-GFPEnvy-T$_{ADH1}$	This study	-	-	pRS316<br>pRS314-P$_{TEF1}$-C-GFPEnvy-T$_{ADH1}$	-
TNP1937	pRS314-P$_{TEF1}$-C-GFP-$\beta_{1–10}$-T$_{ADH1}$	GFP-$\beta_{1–10}$	This study	KM5, KM11	*superfolderGFP* -gBlocks	pRS314-P$_{TEF1}$-C-GFPEnvy-T$_{ADH1}$	-
TNP1938	pRS316-P$_{TEF1}$-C-GFP-$\beta_{1–11}$-T$_{ADH1}$	GFP-$\beta_{1–11}$	This study	KM5, KM15	*superfolderGFP* -gBlocks	pRS316-P$_{TEF1}$-C-GFPEnvy-T$_{ADH1}$	-

S2 Table. Plasmids used for this study (continued).

Number	Name	Protein Expressed/Description	Source	Used Primer	PCR template	Used Plasmid	Figure
TNP1939	pRS316-P_{TEF1}-C-GFP-β_{11}-T_{ADH1}	GFP-β_{11}	This study	KM14, pRS-R	pRS316-P_{TEF1}-C-GFP-β_{1-11}-T_{ADH1}	pRS316-P_{TEF1}-C-GFPEnvy-T_{ADH1}	-
TNP1942	pRS314-mK-SPO20$^{51-91}$-GFP-β_{1-10}-T_{ADH1}	mK-Spo20$^{51-91}$-GFP-β_{1-10}	This study	HT673, HT689<br>HT669, HT678	pRS424-P_{SPR3}-mK-SPO20$^{51-91}$-SAC1$^{2-517}$	pRS314-P_{TEF1}-C-GFP-β_{1-10}-T_{ADH1}	Sup. Fig. 6 B
TNP1941	pRS316-P_{TEF1}-TCB3-GFP-β_{11}-T_{ADH1}	Tcb3-GFP-β_{11}	This study	-	-	pRS316-P_{TEF1}-C-GFP-β_{11}-T_{ADH1}<br>pRS314-P_{TEF1}-TCB3-GFPEnvy	Sup. Fig. 6 B
TNP1801	pRS314-P_{TEF1}-GFPEnvy-OSH1	GFPEnvy-Osh1	This study	TN511, TN512	gDNA of AN120	pRS314-P_{TEF1}-N-GFPEnvy-T_{CYC1}	Sup. Fig. 7
TNP1803	pRS314-P_{TEF1}-GFPEnvy-OSH2	GFPEnvy-Osh2	This study	TN468, TN469	gDNA of AN120	pRS314-P_{TEF1}-N-GFPEnvy-T_{CYC1}	Sup. Fig. 7
TNP1349	pRS314-C-GFPEnvy-T_{ADH1}	C-GFPEnvy-T_{ADH1}	This study	-	-	pRS314-P_{TEF1}-C-GFPEnvy-T_{ADH1}<br>pRS314	-
TNP1805	pRS314-OSH3-GFPEnvy	Osh3-GFPEnvy	This study	TN436, TN437	gDNA of AN120	pRS314-C-GFPEnvy-T_{ADH1}	-
TNP1362	pRS314-P_{TEF1}-C-3×GFPEnvy-T_{ADH1}	P_{TEF1}-C-3×GFPEnvy-T_{ADH}	This study	TN394, TN395<br>TN397, TN414<br>TN422	pRS314-P_{TEF1}-C-GFPEnvy-T_{CYC1}	pRS314-P_{TEF1}-T_{CYC1}	-
TNP1806	pRS314-OSH3-3×GFPEnvy	Osh3-3×GFPEnvy	This study	-	-	pRS314-OSH3-GFPEnvy<br>pRS314-C-3×GFPEnvy-T_{ADH1}	Sup. Fig. 7
TNP1810	pRS314-OSH4-GFPEnvy	Osh4-GFPEnvy	This study	TN438, TN439	gDNA of AN120	pRS314-C-GFPEnvy-T_{ADH1}	Sup. Fig. 7
TNP1813	pRS314-OSH5-3×GFPEnvy	Osh5-3×GFPEnvy	This study	TN523, TN524	gDNA of AN120	pRS314-P_{TEF1}-C-3×GFPEnvy-T_{ADH1}	Sup. Fig. 7
TNP1814	pRS314-OSH6-GFPEnvy	Osh6-GFPEnvy	This study	TN440, TN441	gDNA of AN120	pRS314-C-GFPEnvy-T_{ADH1}	Sup. Fig. 7
TNP1817	pRS314-OSH7-3×GFPEnvy	Osh7-3×GFPEnvy	This study	TN483, TN484	gDNA of AN120	pRS314-P_{TEF1}-C-3×GFPEnvy-T_{ADH1}	Sup. Fig. 7

Reference

1. Christianson TW, Sikorski RS, Dante M, Shero JH, Hieter P. Multifunctional yeast high-copy-number shuttle vectors. Gene. 1992;110: 119–122. doi:10.1016/0378-1119(92)90454-W
2. Nakamura TS, Numajiri Y, Okumura Y, Hidaka J, Tanaka T, Inoue I, et al. Dynamic localization of a yeast development-specific PP1 complex during prospore membrane formation is dependent on multiple localization signals and complex formation. Mol Biol Cell. 2017;28: 3881–3895. doi:10.1091/mbc.E17-08-0521
3. Sikorski RS, Hieter P. A system of shuttle vectors and yeast host strains designed for efficient manipulation of DNA in *Saccharomyces cerevisiae* . Genetics. 1989;122: 19–27. doi:0378111995000377 [pii]
4. Suda Y, Tachikawa H, Inoue I, Kurita T, Saito C, Kurokawa K, et al. Activation of Rab GTPase Sec4 by its GEF Sec2 is required for prospore membrane formation during sporulation in yeast *Saccharomyces cerevisiae* . FEMS Yeast Res. 2018;18: 1–10. doi:10.1093/femsyr/fox095
5. Nakanishi H, Suda Y, Neiman AM. Erv14 family cargo receptors are necessary for ER exit during sporulation in *Saccharomyces cerevisiae* . J Cell Sci. 2007;120: 908–16. doi:10.1242/jcs.03405
6. Longtine MS, McKenzie A, Demarini DJ, Shah NG, Wach A, Brachat A, et al. Additional modules for versatile and economical PCR-based gene deletion and modification in *Saccharomyces cerevisiae* . Yeast. 1998;14: 953–61. doi:10.1002/(SICI)1097-0061(199807)14:10<953::AID-YEA293>3.0.CO;2-U
7. Kamieniecki RJ, Liu L, Dawson DS. FEAR but not MEN genes are required for exit from meiosis I. Cell Cycle. 2005;4: 1093–8. doi:10.4161/cc.4.8.1857
8. Park J-S, Neiman AM. VPS13 regulates membrane morphogenesis during sporulation in *Saccharomyces cerevisiae* . J Cell Sci. 2012;125: 3004–11. doi:10.1242/jcs.105114
9. Roy A, Levine TP. Multiple pools of phosphatidylinositol 4-phosphate detected using the pleckstrin homology domain of Osh2p. J Biol Chem. 2004;279: 44683–44689. doi:10.1074/jbc.M401583200
10. Suda Y, Kurokawa K, Hirata R, Nakano A. Rab GAP cascade regulates dynamics of Ypt6 in the Golgi traffic. Proc Natl Acad Sci U S A. 2013;110: 18976–81. doi:10.1073/pnas.1308627110
11. Stefan CJ, Audhya A, Emr SD. The yeast synaptojanin-like proteins control the cellular distribution of phosphatidylinositol (4,5)-bisphosphate. Mol Biol Cell. 2002;13: 542–57. doi:10.1091/mbc.01-10-0476
12. Janke C, Magiera MM, Rathfelder N, Taxis C, Reber S, Maekawa H, et al. A versatile toolbox for PCR-based tagging of yeast genes: new fluorescent proteins, more markers and promoter substitution cassettes. Yeast. 2004;21: 947–62. doi:10.1002/yea.1142
13. Okumura Y, Nakamura TS, Tanaka T, Inoue I, Suda Y, Takahashi T, et al. The dysferlin domain-only protein, Spo73, is required for prospore membrane extension in *Saccharomyces cerevisiae* . Mitchell AP, editor. mSphere. 2016;1: e00038-15. doi:10.1128/mSphere.00038-15
14. Deng L, Fukuda R, Kakihara T, Narita K, Ohta A. Incorporation and remodeling of phosphatidylethanolamine containing short acyl residues in yeast. Biochim Biophys Acta - Mol Cell Biol Lipids. 2010;1801: 635–645. doi:10.1016/j.bbalip.2010.02.008
15. Park J-S, Thorsness MK, Policastro R, McGoldrick LL, Hollingsworth NM, Thorsness PE, et al. Yeast Vps13 promotes mitochondrial function and is localized at membrane contact sites. Mol Biol Cell. 2016;27: 2435–49. doi:10.1091/mbc.E16-02-0112
