# Supplementary material for: Suppression of Vps13 adaptor protein mutants reveals a central role for PI4P in regulating prospore membrane extension
Source: PLoS Genet. 2021 Aug 18;17(8):e1009727. doi: 10.1371/journal.pgen.1009727 (PMC8372973; doi:10.1371/journal.pgen.1009727)
Supplement: S3 Table — (PDF) [file pgen.1009727.s015.pdf]

S3 Table. Oligonucleotides used for this study.

Name	Sequence (5' → 3')
	**Oligonucleotides for strain construction**
HT5	TACTTTCCTTTCTCCCTGAT
HT403	TTCGAAGGATCTCTTTTCTTC
HT404	TGAGGAGGAAGGTACTAATG
IC8	TGTTCTGCATAATGTCACCT
IC9	GCCTTTACATATACTGTCAC
TN193	GCTTTCGCTCTTAAAGATGAAGAGGCTTTCAATCCTCTGGTAAATCTCATGGCACCCGCTCCAGCGCCTGCACCAGCTCCCAAGTC
TN265	AAATCGATATTTGGATGAAAGATCTTCTTCAAACATACAACTTTTGGAGGTCATCGATGAATTCGAGCTC
TN334	CGAAAACAAGGCACAACAATCAATCTATCGCCCTGTGAGATTTTCAATCTTCATCGATGAATTCGAGCTC
TN335	GATACGAGATCATCCTGTAATACTGGATCTGTGTTTTCCATTTCATTCATGGCACCCGCTCCAGCGCCTG
TN631	CATTCAATATCACAGACGAAAACTCTGGTGAATTAGGAGGAAAAGGCGAGCGCGGCCGCATAGGCCACTA
TN632	CGTTCGCTTTCGCTACTTAAAGAAGCAGGGTCAGGAGAGGGACTTTTGATGGATCCCCGGGTTAATTAAG
TN828	CTGCTAATAGTGCTTTTCAA
TN829	CTGGACTATGGATCGGTATT
TN872	GGACAAAAAATCCGAAGAGAAGCAACGGATCCCCGGGTTAATTAA
TN873	TCCTGCTTAACACTGGAAATGAACAGATATCATCGATGAATTCGA
	**Oligonucleotides for plasmid construction**
HJ47	GAAGAAGAATTAATTAACATGTCCGGTTCTGCTGCTAGTGG
HT669	CTCGAGATGAGGAAAGGCGA
HT673	TGCGGCCGCTTTATGGTGAGCGAGCTGATTAAGG
HT678	CATAAAGCGGCCGCAAAACT
HT689	CTTTCCTCATCTCGAGACCTCCTGCGGATCCACC
HJ48	GAAGAACTCGAGGCTAGTGGATCCGTTCAA
KM5	GAAGAAGAAGCGGCCGCATGAGGAAAGGCGAAGAGCT
KM11	GAAGAAGGCGCGCCTTAATTCAAGTCTTTTGATAATA
KM14	GAAGAACTCGAGGAAAAGAGAGATCACATGGT
KM15	GAAGAAGGCGCGCCTTAAGAAGCATCTGTAATCCCAGCAGCGGTGA
pRS-F	TCACGACGTTGTAAAACGAC
pRS-R	AAACAGCTATGACCATGATT
TN27	GAAGAAGAAGCGGCCGCTTTATGAGTAAAGGAGAAGAACT
TN41	GAAGAAGGATCCATGAGATTTACCAGAGGATT
TN42	GAAGAACTGCAGCTAATCTGAGAAGTACAGTAGAG
TN47	GAAGAACTGCAGCTACATACGAATCAGAGTTAATG
TN49	GAAGAAGGATCCATGCAGTTGTCTATGCGGAT
TN57	GAAGAAGGGCCCAAAACCGGCAGCGATTGGAG
TN64	GAAGAAGAGCTCTCAATCGGATAAACTTCGTT
TN66	GAAGAAGAGCTCAGTAACTCCTGTACTTAACG
TN67	GAAGAAGGTACCATACCGACAGAATTTTCCAA
TN70	GAAGAACTGCAGAGTGGAGGAGAATATTTTAC
TN76	GAAGAACTGCAGATCTGAGAAGTACAGTAGAG
TN78	GAAGAACTGCAGTGAAGTGTTTAACAGATGAG
TN79	GAAGAACTGCAGCTATGAAGTGTTTAACAGATGAG
TN82	GAAGAACTGCAGCTAGAGCAGAGCACCAGTGCCAT
TN95	GAAGAAGAAGCGGCCGCTTTATGGCCTCCTCCGAGGACGTC
TN98	GAAGAACTCGAGGGGTTAATTAACATGGTGAG
TN99	GAAGAAGGTACCAGATCTATATTACCCTGTTATC
TN100	GAAGAAGGATCCACCAGCACTACCTCCAGCAGATCCACCTGCTGAACCTCCGGCAGAACTAGTCTTAGTGGCGTCATC
TN101	GAAGAAGGATCCGCAGGAGGTACAGGTCCAATAGTGTACGTTC
TN102	GGAGAAGGAGAAGTCGACCTAGGGGAATGGCGACTTGATAG
TN105	GAAGAACTGCAGCTAAGAGTTGGGAAACTGGATAAATG
TN106	GAAGAACTGCAGCTAAGCTTGAATCATAAACTTAG
TN116	GAAGAAGGATCCATGTGTGAACAGTATCCCGTTCT
TN119	GAAGAAGAGCTCCTCCTGAGGTGGCAAACAAG
TN120	GAAGAAGGTACCACCACCAGGGACGATGCTGA
TN121	TTCAAGGCTAGACATAATGGTAACAT
TN122	ATGTCTAGCCTTGAATTGCAACAAAT
TN123	GAAGAAGAGCTCCGTGAGTTAAGGTCCATGCA
TN126	GGAGAAGGAGAAGTCGACTCAGTCTTTATAATTTTTCT
TN127	GAAGAAGAGCTCTAAGGGCTACTGTAACCTGC
TN128	GAAGAAGGATCCACCAGCACTACCTCCAGCAGATCCACCTGCTGAACCTCCGGCAGAGGCGCCGGTGGAGTGGCGGC
TN143	TATGGATTCTTTGGACAGAACAAATGTCGTTCAATC
TN144	GAGTTTGTTCTTACAACGGAATGTTGCTCATTAAC
TN145	GAAGAAGGATCCATGCCTAACTCAAATTTTCG
TN146	GAAGAACTCGAGTTAGTAATTGCAATACCTTAG
TN163	GAAGAAGAGCTCTCTATTCCCACCCCAAACTG
TN163	GAAGAAGAGCTCTCTATTCCCACCCCAAACTG

S3 Table. Oligonucleotides used for this study (continued).

Name	Sequence (5' → 3')
TN187	GAAGGAGAATTAATTAACATGATGGGCAGTGTCGAGCTGAATC
TN188	GAAGAAGGCGCGCCGGCACCCGCTCCAGCGCCTGCACCAGCTCCCAAGTC
TN205	GAAGGAGAATTAATTAACATGAAAGAAAAGAGTGCTTGCCC
TN206	GAAGAACTCGAGTTTATACATTCTCAAGTC
TN279	GAAGAAGAGCTCGAATAAAAAACACGCTTTTTCAG
TN280	GAAGAAGAAGCGGCCGCTTTGTTTGTTTATGTGTGTTTATTC
TN289	GGCCAAGGCTGGCCTGACCC
TN290	GGGTCAGGCCAGCCTTGGCC
TN310	GAAGAAAGATCTTCTTTTCGAGCTCCCTAGG
TN311	GAAGGAGAATTAATTAAATAGAAGTATAGTAATTTAT
TN338	GAAGAACTGCAGAGAGTTGGGAAACTGGATAAATG
TN357	ACAAACAAAGCGGCCGCTTTATGGTGAGCGAGCTGATTAA
TN358	CAATTGTCCATTCTAGATCTGTGCCCCAGTTTGCTAG
TN379	GAAGAACTGCAGAGCTTGAATCATAAACTTAG
TN386	GAAGGAGAAGCGGCCGCATAGAAGTATAGTAATTTAT
TN390	GAAGAACTCGAGTACGACTACTTGAATAGATT
TN391	TTGATAGGCAAAAAATCGCA
TN393	GAAGAAGGATCCATGTCAGTCTTGCGATCACA
TN394	GAAGAACTCGAGATGTCTAAAGGCGAGGAATT
TN395	GAAGAAGGCGCGCCTTATTTGTACAATTCGTCCA
TN396	GAAGAAGAAGCGGCCGCTTTATGTCTAAAGGCGAGGAATT
TN397	GAAGAAGGATCCTTTGTACAATTCGTCCATTC
TN408	GAAGAAGAAGCGGCCGCTTTATGTCTGAGCTTATTAAGGA
TN409	GAAGAAGGATCCATTCAGTTTGTGACCAAGTT
TN414	GAAGAAAGATCTTTTATGTCTAAAGGCGAGGAATT
TN419	CTATCAACCCAATCTGTTCT
TN421	GAAGAAGGATCCTTTATGGTGAGCGAGCTGATTAA
TN422	GAAGAAGTCGACTTTGTACAATTCGTCCATTC
TN423	GAAGAAGTCGACTCTGTGCCCCAGTTTGCTAG
TN433	TAAACGCACATAAGCCGACC
TN434	TGCGAGGCCGCCTTTTGCGC
TN436	GAAGAACCGCGGATGAAAATGCAGTGAATTTT
TN437	GAAGAACTCGAGCCAGAGTTGAGAAATATCAG
TN438	GAAGAACCGCGGTACCAGAACCTAATCTTTTA
TN439	GAAGAACTCGAGCAAAACAATTTCCTTTTCTT
TN440	GAAGAAGAGCTCCCCTGCACTGAATTTGTGAA
TN441	GAAGAACTGCAGTTGTTTTGCTGGGTTCTGCT
TN466	GAAGAAGGATCCATGTCCTCCAACCGTGTTCT
TN467	GAAGAAGAATTCTCACATCAAATCAGAAAATC
TN468	GAAGAAGAATTCATGTCTAGGGAAGACTTGTC
TN469	GAAGAACTCGAGTTAAAAAATGTCACCACAAT
TN483	GAAGAAGAGCTCAAAAAAGCATCTAGATAATA
TN484	GAAGAACTCGAGATTCTTTTGGATTCCATGCT
TN490	GAAGAAGAGCTCGCGGGGCCACGAAAAAAAGC
TN491	GAAGAACTCGAGTCTGTAGCACCATCCTAAAA
TN500	GAAGAACTGCAGATGACTGGCATCAAAGCTCA
TN501	GAAGAACTCGAGCTGCGTGTATTCTTGAGGAA
TN511	GAAGAAGGATCCATGGAACAACCTGATCTATC
TN512	GAAGAACTCGAGTTAGAAAATATCAGCACAAT
TN523	GAAGAAGAGCTCATCTTCTCCATTACAGATCA
TN524	GAAGAACTCGAGTATAGTAATTTCGTTCTCCC
TN542	TGGGCCCGGGTATTGTTAGCGGTTTGAAGCAGGCG
TN543	CAATACCCGGGCCCACCACACCGTGTGCATTCGTA
TN550	GAAGAAGAAGCGGCCGCGAGTTAGAAACTATTGATAG
TN633	TGAGAACCAGCTGACTTACT
TN634	TTTTTATATTGCTTTTATTC
TN635	GAAGGAGAACCGCGGTGAGAACCAGCTGACTTACT
TN636	GAAGAAGGTACCTTTTTATATTGCTTTTATTC
TN701	TTACTTTTACGGATCCTAAC
TN702	GTCAGCTGGTTCTCAGTCAA
TN703	AAAGCAATATAAAAAATCCA
TN704	AGAGAAAGCAGGATCCATTT
TN804	CCGCCAGCTGAAGCTTCAGCTGCTAATAGTGCTTTTCAA
TN805	CGCATAGGCCACTAGTCAGCTGGACTATGGATCGGTATT
TN807	ATTGTACAAAGGATCCAACCCTTAATATAACTTCGT
TN810	GACTTTTGATGGATCTACCTAATAACTTCGTATAGCATACA
TN817	TAGAACTAGTGGATCCATGGCCAAAGAAGATACTGG

S3 Table. Oligonucleotides used for this study (continued).

Name	Sequence (5' → 3')
TN818	CTTTAGACATCTCGAGAAACTTAGTTTCTTCTTCGC
TN821	TAGAACTAGTGGATCCATGAGAATAGTGCCAGAAAA
TN822	CTTTAGACATCTCGAGGTAGTATATCCAGCCGACGA
TN823	TAGAACTAGTGGATCCATGACCAGTTTGTCCAAAAG.
TN824	CTTTAGACATCTCGAGACTACCAGAACCTATTAATT
TN830	CCTAACGCTCCAGAAATGCC
TN831	CCTCTTTTTCCGTCATCTTA
YF3	GTGGATCCCCCGGGCTGCAGATGTTAGAGTCTTTAGCTGCTAATT
YF9	CGGGCTGCAGGAATTCATGGCGGATGCCTTTCAA
YF10	TAATTAACCCCTCGAGAGAATGTTTAGAGGCTAGCCT
YF12	GACCTCGAGGGGTTAATTAACATGGTGAGCGAGCTGATTAAGG
YF13	TTTAGAAGTGGCGCGCCTTAACTAGTCTTAGTGGCGTCATCG
YF17	TATAGGGCGAATTGGGTACCATAGCTTCAAAATGTTTCTACT
YF18	GGGAACAAAAGCTGGAGCTCGCGGCCGCAGACATAAAA
YF63	CTTTAGACATCTCGAGTTTCACATCTATGGGTAAATTT
YMSO012	GGAGGAGAGCTCGTGGCGGAGGGGAGATATAC
YMSO013	GGAGGATCTAGATGTATATGAGATAGTTGATT
YMSO017	GGAGGACTCGAGGGAGGTGGAGGTAGTAAAGGAGAAGAACTTTT
YMSO018	TCCTCCGGTACCATATTACCCTGTTATCCCTA
YMSO024	GGAGGACCCGGGGGAGGTGGAGGTATGAGATTTACCAGAGGATT
YMSO025	TCCTCCGTCGACTCAGTACGGAATGCCATTTG
YMSO028	GGAGGAGAGCTCGCTATGGTCCTCCACTGATT
YMSO029	TCCTCCGTCGACAGTGGAGGAGAAAATTTTACC
