# Supplementary material for: Suppression of Vps13 adaptor protein mutants reveals a central role for PI4P in regulating prospore membrane extension
Source: PLoS Genet. 2021 Aug 18;17(8):e1009727. doi: 10.1371/journal.pgen.1009727 (PMC8372973; doi:10.1371/journal.pgen.1009727)
Supplement: Attachment — Submitted filename: review PlosGen 2021.docx [file pgen.1009727.s016.docx]

The work by *Nakamura* et al., entitled *“Suppression of Vps13 adaptor protein mutants reveals a role for PI4P in regulating Vps13 function*” is focused on getting knowledge about the regulation of prospore membrane formation during sporulation process.

Yeast cells possess only one Vps13 protein, but human cells express four different *VPS13* genes *VPS13A-D*, which all are connected to rare neurological diseases. Using the fact that Vps13 in yeast cells influences sporulation the authors study regulation of Vps13 function based on regulation in spores formation. This is an important to get knowledge about regulation of Vps13, a new type of lipid transfer protein, because Vps13 proteins are present in different membrane contact sites thus, their function has to be regulated in time and space.

The important findings of presented manuscript are:

1. Spo71/73 complex requirement, but not Vps13, can be partially overcome by PI4P dephosphorylation
2. Spo73 is required for establishment of membrane contact sites similar to those between ER and PM (marked by the presence of the same tethers) necessary for lipid transfer, but not for Vps13 localization (Figure 7C).
3. In parallel the Vps13 is also necessary to establish the same membrane contact sites as Spo71/Spo73 complex.

**Major concern**

The sporulation is a continuous process, it consists of a series of changes that must occur in the defined order. The presented manuscript uncovers the role of Sec17/73-complex in a context of regulation of prospore membrane formation due to changes in lipid composition influenced by lipid phosphatases, kinases and lipid transfer proteins including Vps13 and regulatory effect of PI4P on the formation of prospore membrane. Thus, the manuscript is not exclusively about Vps13, so author summary do not cover the manuscript content.

The manuscript is clear until the involvement of Vps13 and other tethers in sporulation has been studied. The conclusions drawn from the experiments presented in Figures 7 and 8 and the relevant supplementary materials require further elaboration, so this fragment of the manuscript needs to be refined so that the conclusions are more supported by the results The detailed comments/questions are as follow:

The Spo73 requirement can be overcome by limiting the PI4P level on the PSM. Spo71 binds the membrane by its PH-like domains, then it would be expected that a reduction of PI4P level causes a reduction of Spo71 association with the membrane and this limits Vps13 recruitment. This is consistent with the observation (Figure 7E) that Vps13 does not localize to PSM under suppression conditions. What is the localization of Spo71 in *spo73∆/spo73∆* mutant during sporulation upon suppression condition – limitation of Stt4 action and production of Sac1 targeted to PSM?

The conclusion that Spo71 regulates Vps13 function, drawn from the study in Figure 7, is not convincing. First of all, the suppression by *VPS13* overexpression is extremely low and difficult to judge from Fig. 7B., thus it is difficult to judge that the recruitment of Vps13 to the prosore membrane by a chimeric protein composed of a Spo20 fragment fused to a PxP motif (PxP-K20) is not sufficient for suppression. If this difference is true, however, why does *VPS13* overexpression suppress *spo71∆/spo71∆ spo73∆/spo73∆* deficiency? In this case, there is no Spo71 for recruiting or for regulation of Vps13. There is a possibility that overproduction of Vps13 could suppress the *spo71∆/spo71∆* defect in trans, as in the case when changes in Vps13 localization allow to overcome the ERMES deficiency. In such a case Vps13 recruitment to PSM will not be helpful. This seems very probable as *VPS13* overexpression together with expression of *K20-SEC1-P* has positive additive effect on *spo71∆/spo71∆* *spo73∆/spo73∆* sporulation (Figure 7B) when in *spo71∆/spo71∆* expressing *K20-SEC1-P* (so in very probably in *spo71∆/spo71∆* *spo73∆/spo73∆* as well*)* the Vps13^GFPEnvy is not on PSM (Figure 7E).

A second explanation that the *spo71∆/spo71∆* sporulation defect is not suppressed by PxP-K20 may because Spo71 is required for tethers recruitment (at least Ist2-GFP; Fig 8D, S4C), so recruitment of Vps13 is not enough. The question is, what is the location of the tethers under suppression conditions?

Based on the authors' attempt to suppress the *spo71∆/spo71∆* sporulation defect by targeting Vps13 to PSM via PxP-K20, it can be assumed that the authors believe that Vps13 lipid transfer activity, not localization, is regulated by PI4P. To confirm that PI4P regulates the activity of Vps13 not localization, show Vps13 on the PSM under the conditions as in Figure 7E. Alternatively, test the dynamics of Vps13 (recruitment and dissociation) at PSM.

However, if Spo71 is really necessary for Vps13 to function and not to localize it, why do the authors conclude that it is achieved through PI4P? How does Spo71 affect PI4P that it has a regulatory effect on Vps13? If as authors suggest by Osh proteins please check localization of Osh2/3 in the *spo71∆/spo71∆* mutant.

**Minor points**

1. According to result from Figure 1A it should be impossible to visualize prospore membranes in *spo73∆/spo73∆* cells. Photographs presented in figures clearly present that prospores start to form in this mutant, but are small. What was regarded as spore formation in the studies? Please clarify.
2. Sporulation is a process in which meiosis take place therefore it occurs in diploid cells. However, throughout the manuscript the diploid cells are described as haploids, for example *spo71∆* instead of *spo73∆/spo73∆* etc. This applies to text, figures and figure captions. Please write the first time the *spo73∆/spo73∆* or *spo71∆*/*spo71∆* mutants are used that for simplicity they will be referred as *spo73∆* or *spo71∆*, respectively, throughout the text.
3. Please check carefully whether the abbreviated names of chimeric proteins are clearly named throughout the text and standardize their nomenclature. An exemplary inconsistency in Figure 5B is mKate2-Spo20 but in Figure 5C mKate-Spo20 is truncated to K20.
4. ***Introduction***

Line 72 please give information in the sentence to have the proper order of kinases, the same as the order of their localization.

There are some redundances in an introduction for example lines 83-84 and 91-92 or 107 and 113.

5. ***Results***

The way the sporulation efficiency is counted and results obtained from this studies are presented differs between Figure 1A, 2, 3 and 7A and B – unify

Ad Figure 1D and E – the sporulation (1D) should be tested for the same fragments as interaction (1E). Please provide sporulation rate for fragment Stt4(136-814) instead of three overlapping ones (136-407), (258-498) and (408-814).

Figure 1E The description format is not uniform: -ade and -WLH. Also, there is no information on these letters (WLH) meaning. It should be -A-W-L-H, and later in the figure caption it should be clarified that this is SC medium lacking adenine (A), tryptophan (W), leucine (L) and histidine (H).

In Figure 3A there is ER-VP16 – what is this?

What was measured perimeter of prospore (PS) or of prospore membrane (PSM) (Figure 3)?

Line 245 What do you mean writing: “after incubation for 12 h” What is the starting point for this incubation?

Figure S3 D caption - There is no mKate2-Spo20^51-91^ in figure. Should be: mTagBFP2-Spo20^51-91^, a PSM marker

Please indicate in line 283 which mutant sporulation was analyzed after expression of fusion encoding PxP-K20.

There is no VAB domain described in introduction, but this term is used in results (line 300).

Line 325, please specify what effect on the location of Ist2 was to be tested in the experiment

6. **Discussion**

It is worth to note that not only adaptor proteins, but also lipids are responsible for proper Vps13 localization to different membranes (for example De at al., 2017; Rzepnikowska et al., 2017; Kolakowski et al., 2020).

*L. 368* suppressing the defects of *spo73*∆ - define the type of defect suppressed.

*L. 373* What do you mean “balance of PI4P levels”

*L. 411* What do you mean “acceptor membrane”

Authors noted that “PSM formation begins with the fusion of post-Golgi vesicles on the surface of a proteinaceous matrix, in this case on the spindle pole body (SPB).”, but haven’t that Vps13 could be important for this step. Vps13 interact with Cdc31, a protein of SPB and Vps13-Cdc31 complex is necessary for TGN homotypic fusion (De et al., 2017).

7. **All figures**

The way the figures are marked are not clear. Above the photos there are names of chimeric proteins but sometimes it is written PSM. Please provide in figures the names of extra-genomic constructs transformed into visualized cells. This allows reader to be able to understand the picture without looking at figure caption.

In all cases when overexpression of construct is given please make sure that gene names (capital letters and italics) are provided not proteins (referred to Figures: 2A, 4C, 4D, 5D, 6E, 6F, 7A, 7B, 8C)

8. In a whole manuscript the names of genes and proteins are mixed. Below are the proposed changes.

*Line (L.) 140-141* of these **genes were** constructed

*L. 141* For *EFR3*, deletion **causing lack of** the C-terminal

*L. 144* In the *STT4frag* deletion series, **fragment encoding** Stt4(1–257) was sufficient

*L. 145* capacity of the **Stt4frag and its parts to** bind to

*L. 151* ***STT4frag*** might negatively

*L.* *152* (carrying a D1754A **substitution**)

*L. 153* wild-type ***STT4*** (WT) or ***STT4-KD*** were

*L.* *154* only ***STT4-KD***

*L. 155* overexpression of ***STT4frag*** or ***STT4-KD,* encoding inactive kinase,**

*L. 161* might ***EFR3***

*L. 163* upon **overproduction**

*L. 164* ***EFR3***

*L. 166* **overproduced** Efr3

*L.* *167* overexpression of ***STT4frag*** and ***EFR3*** cause

*L.173 - 174* we repressed the expression of ***STT4*** specifically during sporulation using the promoter of *CLB2* and fusing **sequence coding** an auxin-inducible degron (AID) tag **at** the N-terminus

*L.* *180* expression of ***STT4***

*L. 181* ***GFP-STT4***

*L. 197* suppression, **overproduction** of the (or overexpression of **constructs** **encoding** Sac1-P chimeras)

*L. 205* Although **high level** of Sac1-P chimeras (or overexpression of **constructs** **encoding** Sac1-P chimeras)

*L. 227* expression of **constructs encoding** active

*L. 229* expression of **those encoding** inactive

*L. 235* implicated **in**

*L. 249* tandem PH^PLCδ^ **localization** in

*L. 254* Thus**,**

*L. 257* Co-overexpression of Sac1-P chimera **encoding construct** and ***VPS13***

*L. 259-260* Therefore, we tested the ability of **Sac1 targeted to PSM** to bypass *spo71∆* **mutant sporulation defect**. Indeed**, the presence** of the Sac1-P chimera protein restored **to some extent** **(6.2%)** **the sporulation of** *spo71Δ* diploid

*L. 262 vps13Δ* cells**, even producing the Sac1-P chimera,** did not

*L. 266* of ***VPS13***

*L. 267* when both the Sac1-P chimera **encoding construct** and ***VPS13*** were overexpressed (or when both the Sac1-P chimera and Vps13 were simultaneously **produced at high level** the)

*L. 280* we found that **presence of** **a chimeric protein (called PxP-K20) composed of this domain (Spo71 (359-411)) and the mKate-Spo20^51-91^ (K20) targeting PSM** could restore

*L. 285* PxP-K20 **protein, the presence** of the Sac1-P chimera could not **restore** the localization of Vps13 **to PSM**

*L 287 and 289* overexpression of ***VPS13****.*

*L. 298* **Indeed, the** **fusion protein composed of** extreme N-terminus of Vps13 (1–57) **and** GFP

*L 304* in mutants **lacking** Vps13 **or** the adaptor complex **proteins**.

*L. 305-306* PI4P **level** in the yeast PM **is** regulated in part by the Sac1 phosphatase **through** ER-PM contact sites

*L. 317-318* In this experiment, **simultaneous presence** of mKate-Spo20^51-91^-β1-10 and Tcb3-β11 in wild-type cells **resulted in** GFP fluorescence.

*L. 348* **overproduction** of Vps13

*L. 367* cells **producing** a PI4P-phosphatase
