# Supplementary material for: Suppression of Vps13 adaptor protein mutants reveals a central role for PI4P in regulating prospore membrane extension
Source: PLoS Genet. 2021 Aug 18;17(8):e1009727. doi: 10.1371/journal.pgen.1009727 (PMC8372973; doi:10.1371/journal.pgen.1009727)
Supplement: Attachment — Submitted filename: PLOSGenet rev.docx [file pgen.1009727.s017.docx]

The manuscript „Suppression of Vps13 adaptor protein mutants reveals a role for PI4P in regulating Vps13 function” by Nakamura et al. concerns the role and the regulation of Vps13 protein during sporulation of Saccharomyces cerevisiae. The authors focused on prospore membrane to which Vps13 localizes via the Spo71–Spo73 adaptor complex. They identified multicopy suppressors of *spo73*Δ: a fragment of phosphatidylinositol (PI) 4-kinase catalytic subunit and PI 4-kinase noncatalytic subunit and have found that lowering PI4P levels in the PSM rescues the *spo73*Δ defects. In numerous sophisticated experiments they show that PI4P might regulate Vps13 function and hat Vps13 and the adaptor complex recruit ER-PM tethers to ER-PSM contactsites. They indicated the involvement of PI4P in regulation of Vps13. They also suggested cooperation of distinct contact site of proteins in *de novo* membrane formation. The study is important because it concerns evolutionary conserved lipid transfer protein that function at organelle contact sites and have been implicated in a number of different neurological diseases. The article increases our understanding of mechanisms of remodeling and the function of this important proteins.

The study is well designed and very well executed. It is also excellently written, although the very compact text requires constant attention. My personal wish would be to see similar meticulous research carried out in higher Eukaryota.

Minor comments:

Results

Line 129: explain briefly rationality of ethanol treatment

line 132: include % of sporulation in control parental strain

lines 190-392: it is unclear whether mammalian cells were manipulated with yeast protein, please clarify this fragment

lines 208-210: “Given that the tandem PHOsh2 localizes both on the Golgi and on the PM in budding daughter cells [51], this implies that PHOsh2 preferentially localizes at the PSM rather than the Golgi during PSM formation”

here I do not understand the logic of this conclusion, something seems missing.
