# Supplementary material for: Suppression of Vps13 adaptor protein mutants reveals a central role for PI4P in regulating prospore membrane extension
Source: PLoS Genet. 2021 Aug 18;17(8):e1009727. doi: 10.1371/journal.pgen.1009727 (PMC8372973; doi:10.1371/journal.pgen.1009727)
Supplement: Attachment — Submitted filename: Response to reviewers.docx [file pgen.1009727.s018.docx]

**Responses to the reviewer’s comments**

Reviewer #1

The work by *Nakamura* et al., entitled *“Suppression of Vps13 adaptor protein mutants reveals a role for PI4P in regulating Vps13 function*” is focused on getting knowledge about the regulation of prospore membrane formation during sporulation process.

Yeast cells possess only one Vps13 protein, but human cells express four different *VPS13* genes *VPS13A-D*, which all are connected to rare neurological diseases. Using the fact that Vps13 in yeast cells influences sporulation the authors study regulation of Vps13 function based on regulation in spores formation. This is an important to get knowledge about regulation of Vps13, a new type of lipid transfer protein, because Vps13 proteins are present in different membrane contact sites thus, their function has to be regulated in time and space.

The important findings of presented manuscript are:

1. Spo71/73 complex requirement, but not Vps13, can be partially overcome by PI4P dephosphorylation
2. Spo73 is required for establishment of membrane contact sites similar to those between ER and PM (marked by the presence of the same tethers) necessary for lipid transfer, but not for Vps13 localization (Figure 7C).
3. In parallel the Vps13 is also necessary to establish the same membrane contact sites as Spo71/Spo73 complex.

We thank the reviewer #1 for his/her constructive comments to improve our manuscript.

**Major concern**

The sporulation is a continuous process, it consists of a series of changes that must occur in the defined order. The presented manuscript uncovers the role of Sec17/73-complex in a context of regulation of prospore membrane formation due to changes in lipid composition influenced by lipid phosphatases, kinases and lipid transfer proteins including Vps13 and regulatory effect of PI4P on the formation of prospore membrane. Thus, the manuscript is not exclusively about Vps13, so author summary do not cover the manuscript content.

Following the comment, we modified the author summary to include genetic relation of Vps13 adaptor complex with PI 4-kinase and phosphatase (underlined below):

**Author Summary**

Vps13 family proteins are conserved lipid transfer proteins that function at organelle contact sites and have been implicated in a number of different neurological diseases. In the yeast *Saccharomyces cerevisiae*, Vps13 is encoded by a single gene and is localized to various contact sites by interaction with different adaptor proteins, however its regulation is yet to be clarified. We have previously shown that during the developmental process of sporulation, Vps13 is recruited to *de novo* membrane structures called prospore membranes (PSMs) by a specific adaptor complex, and Vps13 and its adaptors are required for PSM extension. Here we reveal that loss of an adaptor can be overcome by lowering phosphatidylinositol-4-phosphate (PI4P) levels, either by inhibiting PI 4-kinase on the PSM or recruiting PI 4-phospatase to the PSM and that PI4P levels in the PSM affect Vps13 function. Further, we show that Vps13 forms endoplasmic reticulum (ER)-PSM contact sites, that ER-plasma membrane tethering proteins are recruited to ER-PSM contacts, and these proteins may function in conjunction with Vps13. Thus, our work shines light on both the mechanisms of intracellular remodeling and the function of this important class of lipid transfer proteins.

The manuscript is clear until the involvement of Vps13 and other tethers in sporulation has been studied. The conclusions drawn from the experiments presented in Figures 7 and 8 and the relevant supplementary materials require further elaboration, so this fragment of the manuscript needs to be refined so that the conclusions are more supported by the results The detailed comments/questions are as follow:

The Spo73 requirement can be overcome by limiting the PI4P level on the PSM. Spo71 binds the membrane by its PH-like domains, then it would be expected that a reduction of PI4P level causes a reduction of Spo71 association with the membrane and this limits Vps13 recruitment. This is consistent with the observation (Figure 7E) that Vps13 does not localize to PSM under suppression conditions. What is the localization of Spo71 in *spo73∆/spo73∆* mutant during sporulation upon suppression condition – limitation of Stt4 action and production of Sac1 targeted to PSM?

Following the suggestion of reviewer #1, we examined Spo71 localization in the *spo73*∆/*spo73*∆ mutant under suppression conditions. Spo71 localization was not altered by lowering PI4P levels in the PSM. Spo71 localized to the PSM in *spo73∆/spo73∆* P*_CLB2_-degron-stt4/*P*_CLB2_-degron-stt4* cells as in *spo73∆/spo73∆* (Panel A). Also, Spo71 localized to the PSM in *spo73∆/spo73∆* cells overproducing mKate2-Spo20^51-91^-Sac1^2-517^ as in *spo73∆/spo73∆* cells overproducing mKate2-Spo20^51-91^-Sac1^2-517^-PD (Panel B). We note that it is not clear what the ligand(s) of the Spo71 PH domains are *in vivo*. Spo71 may bind lipids other than PI4P or to some protein. Alternatively, because Spo71 contains three PH domains, Spo71 may have high affinity to PI4P on the PSM, which allows localization to the PSM with lowered PI4P. As these data do not add much clarity, we chose not to include them in the manuscript.

As to Fig 7E, we could not detect Vps13 localization to the PSM in *spo71∆/spo71∆* cells overproducing mKate2-Spo20^51-91^-Sac1^2-517^. This result indicates that lowering PI4P in the PSM can weakly suppress *spo71∆/spo71∆* sporulation defect, but this suppression is not by restoring stable localization of Vps13 to the PSM. Though Vps13 localizes to cytoplasm diffusely under these conditions, it is possible that some fraction of Vps13 is still present on the PSM, which would contribute to suppression of s*po71∆/spo71∆* cells by mKate2-Spo20^51-91^-Sac1^2-517^.

The conclusion that Spo71 regulates Vps13 function, drawn from the study in Figure 7, is not convincing. First of all, the suppression by *VPS13* overexpression is extremely low and difficult to judge from Fig. 7B., thus it is difficult to judge that the recruitment of Vps13 to the prosore membrane by a chimeric protein composed of a Spo20 fragment fused to a PxP motif (PxP-K20) is not sufficient for suppression.

As pointed out by reviewer #1, Fig 7B was hard to read. Accordingly, we changed the scale of the y-axis as was also suggested by reviewer #3.

Also, we performed ethanol tests to examine whether spores are formed in s*po71∆/spo71∆* cells producing Spo71^359–411^-mKate2-Spo20^51–91^ (PxP-K20). While s*po71∆/spo71∆* cells carrying empty vector show only a few colonies after ethanol treatment, s*po71∆/spo71∆* cells overproducing Vps13 show 20-50 colonies, indicating existence of spores. As to s*po71∆/spo71∆* cells producing Spo71^359–411^-mKate2-Spo20^51–91^, only a few colonies were formed, indicating no difference to s*po71∆/spo71∆* cells carrying empty vector. Thus, production of Spo71^359–411^-mKate2-Spo20^51–91^ in s*po71∆/spo71∆* cells is not sufficient for sporulation. We added this data as S5C Fig. and changed results as follows (Line 298).

“While *spo71*∆ cells overexpressing *VPS13* produced 20-50 colonies after ethanol treatment, those overproducing this chimera produced only a few colonies, which were similar to those carrying empty vector (S5C Fig).”

Even *spo73∆/spo73∆* cells, in which Spo71-Vps13 localizes properly on the PSM, do not sporulate. In *spo71∆/spo71∆* cells producing Spo71^359–411^-mKate2-Spo20^51–91^, not only domains of the Spo71 are lost, but also interaction with Spo73 is lost. So, it is reasonable that the cells do not sporulate at all.

If this difference is true, however, why does *VPS13* overexpression suppress *spo71∆/spo71∆ spo73∆/spo73∆* deficiency? In this case, there is no Spo71 for recruiting or for regulation of Vps13. There is a possibility that overproduction of Vps13 could suppress the *spo71∆/spo71∆* defect in trans, as in the case when changes in Vps13 localization allow to overcome the ERMES deficiency. In such a case Vps13 recruitment to PSM will not be helpful. This seems very probable as *VPS13* overexpression together with expression of *K20-SEC1-P* has positive additive effect on *spo71∆/spo71∆* *spo73∆/spo73∆* sporulation (Figure 7B) when in *spo71∆/spo71∆* expressing *K20-SEC1-P* (so in very probably in *spo71∆/spo71∆* *spo73∆/spo73∆* as well*)* the Vps13^GFPEnvy is not on PSM (Figure 7E).

As to reviewer #1’s question, why does *VPS13* overexpression suppress *spo71∆/spo71∆ spo73∆/spo73∆* deficiency, we suggest that overproduction increases the population of Vps13 which can transiently access the PSM.

We observed the localization of overproduced Vps13 in WT, *spo73∆/spo73∆*, and *spo71∆/spo71∆* cells also overproducing mKate2-Spo20^51-91^-Sac1^2-517^. In addition to localization to the cytosol and the vacuole, we see PSM pattern in WT and *spo73∆/spo73∆* cells. No apparent PSM pattern was observed in *spo71∆/spo71∆* cells, but Vps13 localizes to cytoplasm diffusely. This observation means some population of Vps13 is accessible to the PSM, which could contribute to suppression. We added this data as S5D Fig. and modified text as follows (Line 302).

“In contrast to Spo71^359–411^-mKate2-Spo20^51–91^, the presence of the Sac1^2–517^ chimera could not restore the localization of Vps13 to the PSM, even when Vps13 was overproduced (Fig 7E and S5D Fig).”

As reviewer #1 suggested, Vps13 could act at some other cellular location than the PSM to suppress *spo71∆/spo71∆* and *spo73∆/spo73∆*. While we cannot rule out this possibility, our new data showing that the Spo71^359–411^-mKate2-Spo20^51–91^ construct, which recruits Vps13 to the PSM, acts synergistically with mKate2-Spo20^51-91^-Sac1^2-517^ to suppress *spo71∆/spo71∆* similar to *VPS13* overexpression, argues that Vps13 functions on the PSM for suppression. We explain this new data below.

A second explanation that the *spo71∆/spo71∆* sporulation defect is not suppressed by PxP-K20 may because Spo71 is required for tethers recruitment (at least Ist2-GFP; Fig 8D, S4C), so recruitment of Vps13 is not enough. The question is, what is the location of the tethers under suppression conditions?

As reviewer #1 suggests, we considered the possibility that restoration of ER-PSM connections through alternate tethers could be the basis of suppression. Ist2-GFP colocalization with the PSM was examined in *spo73∆/spo73∆* and *spo71∆/spo71∆* cells overproducing either WT or phosphatase-defective mKate2-Spo20^51-91^-Sac1^2-517^ (i.e. suppressed or non-suppressed conditions). No significant differences in the fraction of PSMs displaying Ist2-GFP were seen (Left Panel). Thus, increased alternative ER-PSM contacts are not the likely basis for suppression (Added as S6E Fig).

Conversely, it could also be that the failure of Spo71^359–411^-mKate2-Spo20^51–91^ to rescue *spo71∆/spo71∆* is due to a failure to restore alternative ER-PSM contacts. In doing the experiment to examine this point, we noticed that we used a P_TEF1_-SPO71^359–411^-mKate2-SPO20^51–91^ construct in the original experiment. Strong constitutive expression by P*_TEF1_* could affect localization of proteins and have effects on sporulation. Also, in a similar experiment, to produce mKate2-Spo20^51–91^-Sac1^2-517^, we used P*_SPR3_*, which induces the protein specifically at the time of PSM formation. Thus, we decided to redo all the related experiments using a P_SPR3_-SPO71^359–411^-mKate2-SPO20^51–91^ construct. As when produced using P*_TEF1_*, Spo71^359–411^-mKate2-Spo20^51–91^ produced using P*_SPR3_* can recruit Vps13^Envy to the PSM in *spo71∆/spo71∆* cells (Fig 7D, changed), and did not suppress sporulation defect of *spo71∆/spo71∆* at all (Ethanol treatment experiment above). When Ist2-GFP was examined in *spo71∆/spo71∆* cells overproducing either mKate2-Spo20^51–91^ or Spo71^359–411^-mKate2-Spo20^51–91^, the fraction of PSMs showing Ist2-GFP colocalization was unchanged (Center Panel). This suggests that Spo71^359–411^-mKate2-Spo20^51–91^ cannot promote Ist2-GFP localization. However, in these experiments, both cultures showed 65% Ist2-GFP/PSM colocalization, a higher level than seen in *spo71∆/spo71∆* cells producing mKate2-Spo20^51–91^-Sac1^2-517^ where sporulation is seen. It seems unlikely, therefore, that inability to promote Ist2-GFP containing contact sites is the sole reason that Spo71^359–411^-mKate2-Spo20^51–91^ fails to rescue *spo71∆/spo71∆*. These data are now included in S6F Fig.

Surprisingly, we observed that overproduction of Spo71^359–411^-mKate2-Spo20^51–91^ enhanced the rescue of *spo71∆/spo71∆* by mKate2-Spo20^51-91^-Sac1^2-517^. This suggests that the failure of Spo71^359–411^-mKate2-Spo20^51–91^ to rescue *spo71∆/spo71∆* is linked to PI4P levels (Right panel). We have added these data as Fig 7F.

We modified text as follows.

Result

“Moreover, the weak suppression of *spo71*∆ by Sac1^2–517^ was enhanced by the Spo71^359–411^-mKate2-Spo20^51–91^ as by overexpression of *VPS13* (Fig 7F). Thus, while Spo71 recruits Vps13 to the PSM, it must play an additional role in Vps13 function which may be related to PI4P.” (Line 304)

“Notably, the localization of Ist2 was not restored by overproducing Sac1^2–517^ chimera proteins in *spo73*∆ and *spo71*∆ cells (S6E Fig). Further, overproducing Spo71^359–411^-mKate2-Spo20^51–91^ also did not affect Ist2 localization (S6F Fig). These data suggest that suppression of the mutants is not related to restoring the localization of ER-PM tethers.” (Line 352)

Discussion

“Although recruitment of Vps13 to the PSM using PxP motif of Spo71 is not enough for suppression of *spo71*∆, it can enhance suppression of *spo71*∆ by PSM-recruited Sac1^2–517^ chimera, suggesting that PSM-recruited Vps13 is activated by lowering PI4P levels in the PSM.” (Line 398)

“One possibility is that multiple PH domains of Spo71 might be involved in lipid binding for localization of Spo71, for regulation of the Vps13 activity, or both. Consistent with this possibility, co-overexpression of Sac1^2–517^ chimera and Spo71^359–411^ chimera enhanced suppression of *spo71*∆. In this scenario, Spo73 binds Spo71 and could modulate the function of PH domain. For instance, a PH domain of Spo71 might mask PI4P and alleviate the inhibition of Vps13 function.” (Line 449)

Based on the authors' attempt to suppress the *spo71∆/spo71∆* sporulation defect by targeting Vps13 to PSM via PxP-K20, it can be assumed that the authors believe that Vps13 lipid transfer activity, not localization, is regulated by PI4P. To confirm that PI4P regulates the activity of Vps13 not localization, show Vps13 on the PSM under the conditions as in Figure 7E. Alternatively, test the dynamics of Vps13 (recruitment and dissociation) at PSM.

Vps13 localization was confirmed in WT, *spo73∆/spo73∆*, and *spo71∆/spo71∆* cells overproducing mKate2-Spo20^51-91^-Sac1^2-517^. PSM localization in WT and *spo73∆/spo73∆* was not altered by lowering PI4P on the PSM. (*spo71∆/spo71∆* panel: same experiment to Fig 7E) (Left panel)

As to dynamics, we observed Vps13 localization in WT cells with various size of the PSM. Vps13 appears on the PSM when small PSM are formed and localizes on the PSM through PSM formation (Right panel). As these data do not add much clarity, we chose not to include them in the manuscript.

However, if Spo71 is really necessary for Vps13 to function and not to localize it, why do the authors conclude that it is achieved through PI4P? How does Spo71 affect PI4P that it has a regulatory effect on Vps13? If as authors suggest by Osh proteins please check localization of Osh2/3 in the *spo71∆/spo71∆* mutant.

We consider that Spo71 is necessary both for Vps13 localization and Vps13 function. As mentioned above, our new data are consistent with this idea. As to the relation between Spo71 and PI4P, we added following sentence in the discussion as a possibility (Line 449, mentioned above).

“One possibility is that multiple PH domains of Spo71 might be involved in lipid binding for localization of Spo71, for regulation of the Vps13 activity, or both. Consistent with this possibility, co-overexpression of Sac1^2–517^ chimera and Spo71^359–411^ chimera enhanced suppression of *spo71*∆. In this scenario, Spo73 binds Spo71 and could modulate the function of PH domain. For instance, a PH domain of Spo71 might mask PI4P and alleviate the inhibition of Vps13 function.”

Together with the role of Osh proteins we want to clarify these points in near future.

**Minor points**

1. According to result from Figure 1A it should be impossible to visualize prospore membranes in *spo73∆/spo73∆* cells. Photographs presented in figures clearly present that prospores start to form in this mutant, but are small. What was regarded as spore formation in the studies? Please clarify.

Spore formation means the presence of spores visible by light microscopy. This requires completion of the entire sporulation process. Thus, it is possible to have cells that form PSMs but do not generate visible spores. This has been clarified (Line 134).

“To assess the sporulation efficiency, we observed mature spores visible under light microscope.”

2. Sporulation is a process in which meiosis take place therefore it occurs in diploid cells. However, throughout the manuscript the diploid cells are described as haploids, for example *spo71∆* instead of *spo73∆/spo73∆* etc. This applies to text, figures and figure captions. Please write the first time the *spo73∆/spo73∆* or *spo71∆*/*spo71∆* mutants are used that for simplicity they will be referred as *spo73∆* or *spo71∆*, respectively, throughout the text.

We followed the suggestion of reviewer #1. Corrected as follows.

Example

First time: *spo73∆/spo73∆* (hereafter *spo73∆*),

Later: *spo73∆*

3. Please check carefully whether the abbreviated names of chimeric proteins are clearly named throughout the text and standardize their nomenclature. An exemplary inconsistency in Figure 5B is mKate2-Spo20 but in Figure 5C mKate-Spo20 is truncated to K20.

Corrected.

4. ***Introduction***

Line 72 please give information in the sentence to have the proper order of kinases, the same as the order of their localization.

We added “respectively” to clarify the order (Line 75).

There are some redundances in an introduction for example lines 83-84 and 91-92 or 107 and 113.

We deleted explanation of structure from the first part (original line 84) to avoid redundance (Line 87).

We also deleted part of explanation of Spo71 (original line 107) to avoid redundance (Line 111).

5. ***Results***

The way the sporulation efficiency is counted and results obtained from this studies are presented differs between Figure 1A, 2, 3 and 7A and B – unify

Following the suggestion, we modified Fig 1A, 3B, 3D, 4B, 6E and S2B Fig., just to show sporulation efficiency.

Ad Figure 1D and E – the sporulation (1D) should be tested for the same fragments as interaction (1E). Please provide sporulation rate for fragment Stt4(136-814) instead of three overlapping ones (136-407), (258-498) and (408-814).

We did this experiment. The Figure was changed to include the result of sporulation rate for fragment Stt4(136-814).

Figure 1E The description format is not uniform: -ade and -WLH. Also, there is no information on these letters (WLH) meaning. It should be -A-W-L-H, and later in the figure caption it should be clarified that this is SC medium lacking adenine (A), tryptophan (W), leucine (L) and histidine (H).

Following the suggestion, we corrected.

In Figure 3A there is ER-VP16 – what is this?

We added explanation in the legend as follows.

“ER-VP16 is a chimeric protein of the bacterial DNA-binding protein LexA, the hormone-binding domain of the human estrogen receptor (ER) and fragment of the herpes simplex virus type 1 trans-activator VP16. When β-estradiol binds ER, ER-VP16 translocates into nucleus, binds P*_4×lexA_* sequence, and induces expression of downstream coding sequence.

What was measured perimeter of prospore (PS) or of prospore membrane (PSM) (Figure 3)?

We measured perimeter of mKate2-Spo20^51-91^-labeled PSM.

Line 245 What do you mean writing: “after incubation for 12 h” What is the starting point for this incubation?

We changed this to “in post-meiotic cells” (Line 256).

Figure S3 D caption - There is no mKate2-Spo20^51-91^ in figure. Should be: mTagBFP2-Spo20^51-91^, a PSM marker

We thank reviewer #1 for finding this. This is our mistake. Corrected.

Please indicate in line 283 which mutant sporulation was analyzed after expression of fusion encoding PxP-K20.

We added “of *spo71∆*” (Line 298)*.*

There is no VAB domain described in introduction, but this term is used in results (line 300).

We added explanation for VAB domain (Line 292).

“Residues 359–411 of Spo71 contain a “PxP” motif that binds to Vps13 VAB domain”

Line 325, please specify what effect on the location of Ist2 was to be tested in the experiment

We added explanation (Line 343).

“To more carefully examine whether Ist2-GFP signal overlaps with mKate2-Spo20^51-91^ signal,”

6. **Discussion**

It is worth to note that not only adaptor proteins, but also lipids are responsible for proper Vps13 localization to different membranes (for example De at al., 2017; Rzepnikowska et al., 2017; Kolakowski et al., 2020).

We added explanation in discussion as follows (Line 362).

“It is also known that various lipids contribute to proper Vps13 localization to different membranes [20,59,60]”

20: De et al., *J Cell Biol.*, 2017

59: Rzepnikowska et al., *Hum. Mol. Genet.*, 2017

60: Kolakowski et al., *Biochim. Biophys. acta Biomembr.*, 2020

*L. 368* suppressing the defects of *spo73*∆ - define the type of defect suppressed.

We change the text to “suppressing both the PSM extension and sporulation defects of *spo73*∆” (Line 393).

*L. 373* What do you mean “balance of PI4P levels”

As pointed out, these words are not clear and necessary. We deleted these words.

*L. 411* What do you mean “acceptor membrane”

We changed this to “PSM” (Line 436).

Authors noted that “PSM formation begins with the fusion of post-Golgi vesicles on the surface of a proteinaceous matrix, in this case on the spindle pole body (SPB).”, but haven’t that Vps13 could be important for this step. Vps13 interact with Cdc31, a protein of SPB and Vps13-Cdc31 complex is necessary for TGN homotypic fusion (De et al., 2017).

We thank reviewer #1 for this suggestion. In *vps13*∆ cells, small PSMs are formed, so Vps13 is not required for initial fusion of post-Golgi vesicle at the SPB. However, it is still possible that Vps13-Cdc31 can affect efficiency of vesicle fusion at the SPB. We want to check this in near future.

7. **All figures**

The way the figures are marked are not clear. Above the photos there are names of chimeric proteins but sometimes it is written PSM. Please provide in figures the names of extra-genomic constructs transformed into visualized cells. This allows reader to be able to understand the picture without looking at figure caption.

In all cases when overexpression of construct is given please make sure that gene names (capital letters and italics) are provided not proteins (referred to Figures: 2A, 4C, 4D, 5D, 6E, 6F, 7A, 7B, 8C)

Following the suggestions, we modified the Figures.

8. In a whole manuscript the names of genes and proteins are mixed. Below are the proposed changes.

*Line (L.) 140-141* of these **genes were** constructed

*L. 141* For *EFR3*, deletion **causing lack of** the C-terminal

*L. 144* In the *STT4frag* deletion series, **fragment encoding** Stt4(1–257) was sufficient

*L. 145* capacity of the **Stt4frag and its parts to** bind to

*L. 151* ***STT4frag*** might negatively

*L.* *152* (carrying a D1754A **substitution**)

*L. 153* wild-type ***STT4*** (WT) or ***STT4-KD*** were

*L.* *154* only ***STT4-KD***

*L. 155* overexpression of ***STT4frag*** or ***STT4-KD,* encoding inactive kinase,**

*L. 161* might ***EFR3***

*L. 163* upon **overproduction**

*L. 164* ***EFR3***

*L. 166* **overproduced** Efr3

*L.* *167* overexpression of ***STT4frag*** and ***EFR3*** cause

*L.173 - 174* we repressed the expression of ***STT4*** specifically during sporulation using the promoter of *CLB2* and fusing **sequence coding** an auxin-inducible degron (AID) tag **at** the N-terminus

*L.* *180* expression of ***STT4***

*L. 181* ***GFP-STT4***

*L. 197* suppression, **overproduction** of the (or overexpression of **constructs** **encoding** Sac1-P chimeras)

*L. 205* Although **high level** of Sac1-P chimeras (or overexpression of **constructs** **encoding** Sac1-P chimeras)

*L. 227* expression of **constructs encoding** active

*L. 229* expression of **those encoding** inactive

*L. 235* implicated **in**

*L. 249* tandem PH^PLCδ^ **localization** in

*L. 254* Thus**,**

*L. 257* Co-overexpression of Sac1-P chimera **encoding construct** and ***VPS13***

*L. 259-260* Therefore, we tested the ability of **Sac1 targeted to PSM** to bypass *spo71∆* **mutant sporulation defect**. Indeed**, the presence** of the Sac1-P chimera protein restored **to some extent** **(6.2%)** **the sporulation of** *spo71Δ* diploid

*L. 262 vps13Δ* cells**, even producing the Sac1-P chimera,** did not

*L. 266* of ***VPS13***

*L. 267* when both the Sac1-P chimera **encoding construct** and ***VPS13*** were overexpressed (or when both the Sac1-P chimera and Vps13 were simultaneously **produced at high level** the)

*L. 280* we found that **presence of** **a chimeric protein (called PxP-K20) composed of this domain (Spo71 (359-411)) and the mKate-Spo20^51-91^ (K20) targeting PSM** could restore

*L. 285* PxP-K20 **protein, the presence** of the Sac1-P chimera could not **restore** the localization of Vps13 **to PSM**

*L 287 and 289* overexpression of ***VPS13****.*

*L. 298* **Indeed, the** **fusion protein composed of** extreme N-terminus of Vps13 (1–57) **and** GFP

*L 304* in mutants **lacking** Vps13 **or** the adaptor complex **proteins**.

*L. 305-306* PI4P **level** in the yeast PM **is** regulated in part by the Sac1 phosphatase **through** ER-PM contact sites

*L. 317-318* In this experiment, **simultaneous presence** of mKate-Spo20^51-91^-β1-10 and Tcb3-β11 in wild-type cells **resulted in** GFP fluorescence.

*L. 348* **overproduction** of Vps13

*L. 367* cells **producing** a PI4P-phosphatase

We thank reviewer#1 for pointing these out. Following the suggestions, we changed the text.

Reviewer #2

The manuscript „Suppression of Vps13 adaptor protein mutants reveals a role for PI4P in regulating Vps13 function” by Nakamura et al. concerns the role and the regulation of Vps13 protein during sporulation of Saccharomyces cerevisiae. The authors focused on prospore membrane to which Vps13 localizes via the Spo71–Spo73 adaptor complex. They identified multicopy suppressors of spo73Δ: a fragment of phosphatidylinositol (PI) 4-kinase catalytic subunit and PI 4-kinase noncatalytic subunit and have found that lowering PI4P levels in the PSM rescues the spo73Δ defects. In numerous sophisticated experiments they show that PI4P might regulate Vps13 function and hat Vps13 and the adaptor complex recruit ER-PM tethers to ER-PSM contactsites. They indicated the involvement of PI4P in regulation of Vps13. They also suggested cooperation of distinct contact site of proteins in de novo membrane formation. The study is important because it concerns evolutionary conserved lipid transfer protein that function at organelle contact sites and have been implicated in a number of different neurological diseases. The article increases our understanding of mechanisms of remodeling and the function of this important proteins.

The study is well designed and very well executed. It is also excellently written, although the very compact text requires constant attention. My personal wish would be to see similar meticulous research carried out in higher Eukaryota.

We thank the reviewer #2 for his/her positive comments.

Minor comments:

Results

Line 129: explain briefly rationality of ethanol treatment

We added the explanation in the text (Line 131).

“Only mature spores are resistant to ethanol, while vegetative cells and immature or aberrant spores are sensitive.”

line 132: include % of sporulation in control parental strain

As suggested, we modified the text to explain *spo73∆* mutant does not sporulate at all (Line 135).

lines 190-392: it is unclear whether mammalian cells were manipulated with yeast protein, please clarify this fragment

Correct. Mammalian cells were manipulated with yeast protein. To clarify this, we added the word “heterologously” in the text (Line 201).

lines 208-210: “Given that the tandem PHOsh2 localizes both on the Golgi and on the PM in budding daughter cells [51], this implies that PHOsh2 preferentially localizes at the PSM rather than the Golgi during PSM formation”

here I do not understand the logic of this conclusion, something seems missing.

Sorry for confusing explanation. We rewrote this part as follows (Line 218).

“No Golgi localization of the reporters was seen in sporulating cells. Given that the tandem PH^Osh2^ localizes both on the Golgi and on the PM in the bud during vegetative growth [51], exclusive localization of PH^Osh2^ to the PSM implies that PH^Osh2^ has high affinity to the PSM during PSM formation.”

Reviewer #3:

Nakamura et al. used genetic approach accompanied by a wide set of cytological tools to study the role of the conserved lipid transfer protein Vps13 in formation of the prospore membrane (PSM) during sporulation in budding yeast. The paper presents several novel findings that are well supported by the experimental data. However, in my opinion, the major conclusion of the paper that phosphatidylinositol-4-phosphate (PI4P) levels regulate activity of Vps13 at the PSM seems a little far-fetched. There is a clear interplay between the PI4P content in the PSM and the function of Vps13 complex; however, the mechanism of Vps13 regulation by PI4P has not been elucidated mechanistically and the influence of PI4P on Vps13 may also be indirect. In the discussion, the authors themselves proposed an alternative interpretation of their results pointing to the possible involvement of PI levels instead of PI4P in Vps13 regulation (page 13, lines 431-437).

The experimental work is generally well-done and described. However, several issues need to be addressed or clarified before acceptance. I also suggest some changes in figure layouts and abbreviations to improve the perception of content.

We thank the reviewer #3 for his/her positive comments.

Major points:

The title does not reflect well major findings of the paper. The title should rather emphasize the role of Vps13 in the establishment of the ER-PSM contact sites and demonstration of high PI4P levels in the PSM, both of which are essential for extension and maturation of the PSM.

We changed the title to explain the content more broadly.

*“Suppression of Vps13 adaptor protein mutants reveals a central role for PI4P in regulating prospore membrane extension”*

Is there any effect of STT4frag, STT4, STT4-KD and EFR3 overexpression on sporulation efficiency in wild-type background?

Following the suggestion, we performed this experiment. The result is as follows. *EFR3* overexpression affected sporulation of WT cells.

The result is interesting, but as these data are off topic, we chose not to include them.

Overexpression of STT4-KD in the spo73 mutant resulted in 4.3% sporulation efficiency, whereas spo73 cells overexpressing STT4frag showed 4 times higher sporulation rate. Both Stt4 variants lack kinase activity and should show similar levels of suppression. Please comment this discrepancy.

Figure 1C: it is shown that overexpression of 651-782 fragment of Efr3 in the spo73 mutant resulted in 2-fold higher sporulation efficiency compared to full length Efr3. Please comment this discrepancy.

To these comments of the reviewer #3, we added the following sentence in the text (Line 154).

“The degree of suppression varied somewhat between different *STT4* and *ERF3* constructs, likely due to differences in expression level, though this has not been directly examined.”

Page 6, line 183: it is stated that “the downregulation of Stt4 directly suppressed the defect of spo73del (Fig. 3 D)”. Can suppression, as a genetic term, be direct or indirect?

We thank the reviewer #3 for pointing this out. We deleted “directly”.

Figures 4D and 6F lack representative photos showing different perimeters of PSM in indicated strains.

Following the suggestion, we added representative photos to Fig 4C and 6F.

Page 8, lines 241-243, 248-249 and Figure 6B: what was the purpose of checking PI3P distribution in spo73 vs wild-type cells?

PI3P is a major PI moieties in yeast in addition to PI4P and PI(4,5)P_2_ which can be detected with biomarkers. So, we considered it informative. Also, it is reported that Spo71 PH domains interact with PI3P in vitro (Yu JW et al. 2004 Molecular cell Vol.13 677-688). Thus, we wanted to check its distribution.

Figure 7B: what is the effect of Vps13 and K20-Sac1-P on sporulation efficiency in spo71 and spo73 single mutants? Also, what is the localization pattern of Vps13 when overexpressed in spo mutants? Does it show any colocalization with PSM?

*spo73∆* was suppressed significantly by overproduction of Vps13 and mKate2-Spo20^51-91^-Sac1^2-517^ (K20-Sac1-P). Suppression of *spo71∆* was similar level to *spo71∆ spo73∆* mutant. We included this as the S5A and B Fig.

As to localization pattern of Vps13, we observed overproduced Vps13 localization in WT, *spo71*∆, and *spo73*∆ cells. In addition to localization to the cytosol and the vacuole, we see PSM pattern in WT and *spo73*∆ cells. No apparent PSM pattern was observed in *spo71*∆ cells, but Vps13 localizes to cytoplasm diffusely, which means some population of Vps13 is accessible to the PSM, which could contribute to suppression. As these data do not add much clarity, we chose not to include them in the manuscript.

Figure layouts:

Figure 1A, 2A, 3B, 3D, 4C and 6E: first, is it necessary to show percentage of all ascus classes observed? The authors never referred to these classes in the text. In my opinion, showing just the total percentage of asci having at least one spore is sufficient. Second, I suggest graphs showing sporulation efficiency (%) in the way presented in Figures 7A and 7B and the maximum value of Y axis does not need to be 100% (same for Fig. 7A and 7B). This would help to read the % values in Figures without referring to the text.

I suggest to combine Figure 2A and Figure S1A into a separate supplemental figure.

I suggest to incorporate panels Fig.S1B and Fig.S1E into panels Fig.2B and Fig.2C, respectively. The reader of the present version has to jump back and forth between Fig.2 and Fig.S1.

Figure 4: I suggest to present panel B as a supplemental figure.

We thank reviewer #3 for comments on the Figure layout. We followed the suggestions.

Abbreviations:

Some abbreviations of chimeric proteins are confusing and I suggest to stick to full versions both in the text and Figures for nomenclature consistency and overall clarity of the content.

- “Sac1-P” may also refer to phosphorylated form of the protein; I suggest using “Sac1<2-517>superscript”

- “R20” may also refer to Arg20; I suggest to use always the full name “mRFP-Spo20<51-91>superscript”

- Use 3xmKate2-Spo20<51-91>-Sac1<2-517> instead of “3xK20-Sac1-P” as “K20” may also refer to Lys20 and such abbreviation may indicate three copies of both mKate2 and Spo20.

- Use “Spo71<359-411>superscript” instead of “PxP-K20”.

We thank reviewer #3 for pointing these out. Following the suggestions, we changed abbreviations.

Minor points:

Page 3, line 92: correct citation style.

Corrected (Line 94).

Page 5, line 129: provide rationale behind isolation of ethanol-resistant spores.

We added the explanation for ethanol treatment (Line 131).

“Only mature spores are resistant to ethanol, while vegetative cells and immature or aberrant spores are sensitive.”

Legend of Figure 1E: Stt4(136-814) and Efr3(651-782) were used a control. A control of what? Please explain in the legend.

We are sorry for confusing explanation.

“Binding of Ypp1 and a Efr3 fragment was assessed as a positive control for functionality of GAD-Ypp1”.

Legend of Figure S2, panel B: localization of GFP-P4M is shown during vegetative growth not during PSM formation as described in the legend.

Legend of Figure S2, panel C: localization of GFP-P4M is shown during PSM formation not during vegetative growth as described in the legend.

We thank reviewer #3 for pointing this out. Corrected.

Please describe the mRFP-Spo2051-91 fusion protein when first mentioned on page 5, line 159. First description is on page 6, lines 193-194.

We added explanation (Line 167).

“mRFP fused to the sequence of Spo20 which binds phosphatidic acid on the PSM”

Please explain in the text what are Sfp1 (page 5, line 159), pik1 ts mutant (page 7, line 221), gip1del (page 10, line 323) and why they were tested.

We added explanations.

Sfk1, a transmembrane protein at the PM which is reported to bind to Stt4 [41] (Line 168)

Pik1: *PIK1*, which encodes Golgi-localized PI4K (Line 230)

*gip*1∆: To examine whether alteration of Ist2 localization is caused by PSM extension defect, we observed Ist2 localization in *gip1*Δ/*gip1*Δ (hereafter *gip1*Δ) cells, defective in PSM extension by a distinct mechanism (Line 339).

Page 7, lines 219-221: instead of saying that “localization was altered”, please describe in detail localization changes of Osh2-P4M in stt4-4 and pik1-83 cells (Figure S3C).

We added more detailed explanation (Line 229).

“To examine dependency of Osh2-P4M localization on PI4P, we used temperature-sensitive (ts) mutants of *STT4* and *PIK1*, which encodes Golgi-localized PI4K. When *stt4-ts* and *pik1-ts* are incubated at nonpermissive temperature, the PI4P pool of the PM and the Golgi are depleted, respectively. Under this condition, Osh2-P4M did not localize to the PM in *stt4-ts*, and to the Golgi in *pik1-ts* (S4C Fig).”

Figure 9 should be titled “Hypothetical model..”.

Following the suggestion, we changed the Figure title.

We thank all the reviewers for reviewing our manuscript.
