# Supplementary material for: Suppression of Vps13 adaptor protein mutants reveals a central role for PI4P in regulating prospore membrane extension
Source: PLoS Genet. 2021 Aug 18;17(8):e1009727. doi: 10.1371/journal.pgen.1009727 (PMC8372973; doi:10.1371/journal.pgen.1009727)
Supplement: Attachment — Submitted filename: Responses to the reviewer 2nd_20210719.docx [file pgen.1009727.s019.docx]

**Responses to the reviewer’s comments**

Reviewer #1

The manuscript is now greatly improved, according to reviewers suggestions.

We thank reviewer 1 for his/her constructive comments in the last revision.

However, we suggest minor corrections which are listed below.

The authors added in the discussion that Vps13 may be recruited to membranes due to interactions with lipids, in addition to interactions with proteins, but this information should also be included on line 57.

As suggested by the reviewer, we added this explanation.

“by interaction with different adaptor proteins and/or lipids,“ (Line 56-57)

The conclusion in P10,L306 should be rather extended to point the other possibility: “and enhanced suppression by overexpression of VPS13 may depend on Vps13 function in other localisation than PSM.”

While we thank the reviewer for suggestion, P10, L306 is not suitable for placing this sentence. So, we added the following sentence to L284-285.

“Enhanced suppression by overexpression of *VPS13* could also depend on Vps13 function in other localization than the PSM.”

Figure 7

Inconsistency in panels, in C Vps13 ^ Envy and mK-Spo20 51-91, but in D,E and G fusion proteins are listed above the line and pictures are labelled GFP and mK.

To standardize, mKate in panel G should be shortened to mK, as in the rest of the text. It is not known from the figure which protein tagged with GFP is shown in G.

We thank the reviewer for pointing these out. We corrected Figure 7 C and G.

“Construct” is a laboratory language. Better use terms: plasmid, gene, gene fusion, instead (L239, 280).

As suggested by the reviewer, we corrected as follows.

“Upon the expression of gene fusions encoding” (Line 240)

“chimera encoding gene fusion construct and” (Line 281)

P4, L127 Overexpression of EFR3 or an fragment of STT4 gene
P5, L159 we constructed a plasmid encoding a kinase-dead (KD) Stt4…. based on the mutant Pik1 (or pik1 mutant)
P7, L203, we constructed genes encoding fused
L2-4, PSM, or Dtr1.
P9, L269 chimera encoding gene fusion
P14, L443, Vps13 overproduction
L451, co-overproduction
P28, L845 Overexpression of EFR3 or truncated STT4
P29, L88, add “The” to avoid starting the sentence with small letter.
L30, L916 markers used
P31, L936 Wild-type

We thank reviewer 1 for pointing these out. Following suggestions, we corrected.
